# Have there been sustained impacts of the COVID-19 pandemic on trends in smoking prevalence, uptake, quitting, use of treatment, and relapse? A monthly population study in England, 2017–2022

**DOI:** 10.1186/s12916-023-03157-2

**Published:** 2023-12-14

**Authors:** Sarah E. Jackson, Harry Tattan-Birch, Lion Shahab, Emma Beard, Jamie Brown

**Affiliations:** 1 Department of Behavioural Science and Health, University College London, 1-19 Torrington Place, London, WC1E 7HB UK; 2 SPECTRUM Consortium, London, UK; 3 Department of Epidemiology and Public Health, University College London, 1-19 Torrington Place, London, WC1E 7HB UK

**Keywords:** COVID-19, Smoking, Cessation, Quit attempts, Behavioural support, E-cigarettes, Varenicline, Nicotine replacement therapy

## Abstract

**Background:**

Studies conducted during the early stages of the pandemic documented mixed changes in smoking behaviour: more smokers quitting successfully but little change in prevalence. This study aimed to examine whether there have been sustained impacts of the COVID-19 pandemic on smoking patterns in England.

**Methods:**

Data were from 101,960 adults (≥ 18 years) participating in the Smoking Toolkit Study, a monthly representative household survey, between June 2017 and August 2022. Interviews were conducted face-to-face until March 2020 and via telephone thereafter. Generalised additive models estimated associations of the pandemic onset (March 2020) with current smoking, uptake, cessation, quit attempts, and use of support. Models adjusted for seasonality, sociodemographic characteristics, and (where relevant) dependence and tobacco control mass-media expenditure.

**Results:**

Before the COVID-19 pandemic, smoking prevalence fell by 5.2% per year; this rate of decline slowed to 0.3% per year during the pandemic (RR_Δtrend_ = 1.06, 95% CI = 1.02, 1.09). This slowing was evident in more but not less advantaged social grades (RR_Δtrend_ = 1.15, 1.08, 1.21; RR_Δtrend_ = 1.00, 0.96, 1.05). There were sustained step-level changes in different age groups: a 34.9% (95% CI = 17.7, 54.7%) increase in smoking prevalence among 18–24-year-olds, indicating a potential rise in uptake, in contrast to a 13.6% (95% CI = 4.4, 21.9%) decrease among 45–65-year-olds. In both age groups, these step-level changes were followed by the pre-pandemic declines stopping, and prevalence remaining flat. There were sustained increases in quitting among past-year smokers, with a 120.4% (95% CI = 79.4, 170.9%) step-level increase in cessation and a 41.7% (95% CI = 29.7, 54.7%) increase in quit attempts. The main limitation was the change in modality of data collection when the pandemic started; while this may have contributed to the step-level changes we observed, it is unlikely to explain changes in the slope of trends.

**Conclusions:**

In England, the rate of decline in adult smoking prevalence stagnated during the COVID-19 pandemic through to 2022. At the start of the pandemic, a potential reduction in smoking prevalence among middle-aged adults and increases in quitting among smokers may have been offset by an increase in smoking among young adults. The slowing in the rate of decline was pronounced in more advantaged social grades.

**Supplementary Information:**

The online version contains supplementary material available at 10.1186/s12916-023-03157-2.

## Background

The COVID-19 pandemic had a profound impact on everyday life, public health, and health services. Studies conducted during the early stages of the pandemic documented mixed changes in smoking behaviour. Many observed short-term increases in rates of quit attempts and cessation among smokers [1–4], indicating the pandemic may have prompted smokers to stop. However, surveys that measured smoking prevalence produced inconsistent findings (both between and within countries), including increases, decreases, and no substantial change in the proportion of adults who smoke [1, 2, 5]. Evidence on changes in smoking prevalence has been limited by many studies using non-representative samples [2] and nationally representative surveys undergoing substantial changes to their methods of data collection and sample weighting as a result of social distancing restrictions [5]. Relatively little is known about what impact the pandemic has had on uptake of smoking [2, 6], relapse among ex-smokers, or use of support by smokers trying to quit [1] (see Additional File 1 for a more detailed literature review [1–3, 5–7]).

Identifying whether any short-term changes in smoking patterns following the onset of the pandemic have translated into long-term, sustained changes, and the groups in which they have occurred, is essential for building a clear picture of its public health impact and targeting policy, messaging, and support services. As highlighted by Sarich et al. [2], there is some evidence that lifestyle behaviours adopted during a pandemic can persist for some time — for example, sustained increases in alcohol abuse/dependence symptoms were observed three years after the 2003 severe acute respiratory syndrome outbreak among individuals in China who were quarantined or worked in high-risk settings during the epidemic [8]. On the other hand, it is possible that once life started to return to ‘normal’ after the early months of the pandemic, people reverted to their previous smoking patterns and quitting became a less salient issue.

Two and a half years on from the start of the pandemic, there were sufficient data within the Smoking Toolkit Study (a representative monthly survey of adults in England) to undertake a more detailed analysis of whether there has been a sustained impact of the COVID-19 pandemic on smoking patterns. In collecting data monthly, the Smoking Toolkit Study affords a unique opportunity to assess detailed trends at this stage (most representative surveys collect these data at much less frequent intervals). Specifically, we aimed to address the following research questions. Using data from June 2017 through August 2022:

1. What has been the sustained impact of the COVID-19 pandemic on monthly trends in:
  - Current smoking (among all adults);
  - Current smoking among young adults (to assess uptake of smoking);
  - Current smoking among middle-aged adults (to gauge late relapse);
  - Cessation and making ≥ 1 serious quit attempt (among past-year smokers);
  - Number of past-year quit attempts and use of cessation support in the most recent attempt (among past-year smokers who made ≥ 1 quit attempt)?

We also explored the impact of the COVID-19 pandemic on these outcomes separately by socioeconomic position.

## Methods

### Pre-registration

The analysis plan (Additional File 2) was pre-registered on Open Science Framework (https://osf.io/vy254/). We made one amendment prior to peer review: the model assessing medium-term relapse (i.e. failure of quit attempts that started 6–12 months prior to the survey) had problems with convergence due to a very high rate of relapse (83.0%), so we excluded this outcome. Following comments from reviewers, we analysed cessation and quit attempts separately for those aged 18–24 and ≥ 25, to explore whether any changes in these outcomes differed by age.

### Design

The Smoking Toolkit Study uses a hybrid of random probability and simple quota sampling to select a new sample of 1700 adults representative of the adult population in England each month [9]. Interviews are held with one household member in selected geographic output areas until quotas are fulfilled. The quotas are based on factors influencing the probability of being at home (i.e. working status, age and gender). This hybrid form of random probability and quota sampling is considered superior to conventional quota sampling. Here, the choice of households to approach is limited by the random allocation of small output areas and rather than being sent to specific households in advance, interviewers can choose which households within these small geographic areas are most likely to fulfil their quotas. Therefore, unlike random probability sampling, it is not appropriate to record the response rate in the Smoking Toolkit Study. Comparisons with sales data and other national surveys indicate that key variables including sociodemographics, smoking prevalence, and cigarette consumption are nationally representative [9, 10].

Data were collected monthly, initially through face-to-face computer-assisted interviews. However, social distancing restrictions under the COVID-19 pandemic meant no data were collected in March 2020 and data from April 2020 onwards were collected via telephone. The telephone-based data collection used the same combination of random location and quota sampling, and weighting approach as the face-to-face interviews and comparisons of the two data collection modalities indicate good comparability [1, 11, 12]. Nonetheless, it will not be possible to determine with certainty whether any step-level changes (i.e. abrupt shifts in the prevalence of a given outcome) observed are due to the pandemic or the switch from face-to-face to telephone interviewing. While step-level changes may have been affected, changes in the *slope* of trends from before to after the pandemic are likely unaffected — given that there were no further updates in methodology after April 2020.

For the present study, we used individual-level data collected between June 2017 and August 2022. We selected June 2017 as the first month of data for this analysis because it provided a period with a relatively stable tobacco control climate in England (following the implementation of the Tobacco Products Directive between May 2016 and May 2017) meaning the effects of the pandemic on smoking outcomes could more easily be detected. August 2022 provided a sensible end point because it was a time when COVID-19 was still considered a global emergency [13] but was before interest rates increased substantially [14] (changes in smoking behaviour beyond this point may have been more affected by the cost-of-living crisis than the pandemic). Because the sample was restricted to people aged ≥ 18 years when data collection switched from face-to-face to telephone interviews, we excluded any participants aged 16–17 recruited before April 2020 for consistency.

This study is reported as per the Strengthening the Reporting of Observational Studies in Epidemiology (STROBE) guideline (Additional File 3).

### Measures

Full details of the measures (including question wording and derivation) are provided in Additional File 4 [15–20].

We assessed the following outcomes:

- Among all adults: *current smoking* (any type of tobacco);
- Among 18–24-year-olds: current smoking (as an indicator of *uptake*, because any increases in this age group would largely be driven by uptake rather than relapse [7, 21]);
- Among 45–65-year-olds: current smoking (as an indicator of *late relapse*, because an increase in this age group would largely be driven by relapse rather than uptake — on the basis that very few people take up smoking in later life [7]. The upper age limit for this group was selected to minimise any impact of increased mortality at older ages during the pandemic on smoking prevalence);
- Among past-year smokers: *cessation* (coded 1 for those who reported having stopped smoking completely in the last year and 0 for those who reported being a current smoker) and *making* ≥ *1 serious quit attempt* in the past year;
- Among past-year smokers who made ≥ 1 quit attempt in the past year: *number of quit attempts* made (log-transformed), *use of prescription medication* (varenicline/bupropion/nicotine replacement therapy), *use of behavioural support* (face-to-face support/telephone support/websites/apps/written self-help materials), and *use of e-cigarettes* (note that in England, e-cigarettes are recommended as a smoking cessation aid [22] and are available to purchase without a prescription).

Covariates included age, gender, occupational social grade (ABC1 = managerial/professional/intermediate, C2DE = small employers/lower supervisory/technical/semi-routine/routine/never workers/long-term unemployed), region in England, and (where relevant) level of dependence and government spending on tobacco control mass media campaigns.

### Statistical analyses

Data were analysed in R v.4.2.1 [23]. Missing cases were excluded on a per-analysis basis. We calculated unweighted and weighted descriptive statistics on sociodemographic and smoking characteristics. The Smoking Toolkit Study uses raking to match the sample to the population in England on the dimensions of age, social grade, region, housing tenure, ethnicity, and working status within sex [9]. All the following analyses were done on weighted data.

We used segmented regression to assess the effect of the onset of the COVID-19 pandemic on each outcome. We chose this approach over a more detailed analysis of how trends have varied during different periods of the pandemic because sustained, long-term changes have greater relevance to tobacco control policy in England. We used log-binomial generalised additive models (GAMs). These allow the fitting of smoothing terms (e.g. cyclic cubic splines) to take seasonality into account. We modelled the trend in each outcome before the pandemic (underlying secular trend; coded 1…*n*, where *n* was the total number of waves), the step-level change (coded 0 before the start of the pandemic in March 2020 and 1 after), and change in the trend (slope) post-onset of the pandemic relative to pre-pandemic (coded 0 before the pandemic and 1…*m* from April 2020 onwards, where *m* was the number of waves after the start of the pandemic). Models were adjusted for seasonality (modelled using a smoothing term with cyclic cubic splines specified) and covariates. A linear pre-pandemic and pandemic trend was assumed, based on prior data [24] and the relatively short length of the time-series (meaning we expected negligible differences between log-linear and linear trends). We repeated models separately by social grade (ABC1/C2DE). We also repeated the models for cessation and quit attempts separately for 18–24-year-olds and ≥ 25-year-olds to explore differences by age. We used predicted estimates from these models to plot time trends in the weighted prevalence (or mean, for the number of quit attempts) of each outcome alongside unadjusted, weighted monthly data points.

Planned sensitivity analyses tested for pulse effects (i.e. short-lived changes in our outcomes at the start of the pandemic), to explore the possibility that any changes detected in our primary models better reflected transient (vs. sustained) impacts of the pandemic. We ran GAMs with pulses lasting two and three months (coded 0 before the start of the pandemic, 1 in the two or three months after the onset of the pandemic, and 0 thereafter), assuming a constant underlying time trend. Next, we reran models for cessation and use of cessation support excluding our measure of cigarette dependence (strength of urges to smoke) as a covariate, because this could plausibly have been affected by the COVID-19 pandemic (e.g. increased due to stress or reduced due to less exposure to others smoking) and thus adjusting for it may have served to dilute the true impact of the pandemic on these outcomes. We also reran the model for the use of prescription medication excluding varenicline, to check whether the results were affected by the unavailability of this medication from mid-2021 due to manufacturer recall.

Finally, we included an unplanned analysis in which we modelled changes in cigarette dependence in relation to the COVID-19 pandemic (using GAMs as described above, with adjustment for age, gender, social grade, and region), to provide context on differences between analyses that did and did not include dependence as a covariate.

## Results

There were 102,371 respondents to the Smoking Toolkit Study between June 2017 and August 2022. We excluded 411 people (0.4%) who did not report their smoking status, leaving a sample of 101,960 for analysis. Of these, 55,349 were surveyed before the start of the pandemic (June 2017–February 2020) and 46,611 were surveyed during the pandemic (April 2020–August 2022). There was a small proportion of missing cases on quitting outcomes (4.1% for quit attempts; 0% for cessation, number of quit attempts, and use of support). Table 1 presents weighted descriptive statistics for the sample as a whole and as a function of the timing of the pandemic (unweighted characteristics are shown in Additional File 5: Table S1).

**Table 1:** Descriptive statistics

	Overall	Before pandemic (June 2017–Feb 2020)	During pandemic (April 2020–Aug 2022)	p*
*Unweighted N*	*101,960*	*55,349*	*46,611*	
Age (years)	48.3 (18.7)	47.8 (18.9)	48.7 (18.6)	< 0.001
18–24	12.1%	12.8%	11.3%	-
25–44	33.2%	33.1%	33.3%	-
45–65	33.2%	33.0%	33.5%	-
> 65	21.5%	21.1%	21.9%	-
Gender				< 0.001
Men	48.9%	49.0%	48.7%	-
Women	50.8%	50.9%	50.7%	-
In another way	0.3%	0.1%	0.6%	-
Social grade C2DE (less advantaged)	44.2%	44.4%	43.9%	0.178
Region in England				0.889
London	15.5%	15.5%	15.5%	-
South	26.5%	26.4%	26.5%	-
Central	30.2%	30.2%	30.3%	-
North	27.8%	27.9%	27.7%	-
Current smoker				
All adults	16.8%	17.0%	16.6%	0.147
Young adults^1^	22.7%	21.7%	24.0%	0.006
Middle-aged adults^2^	15.2%	16.5%	13.5%	< 0.001
Past-year smoker	18.5%	18.1%	19.0%	0.001
Cigarette dependence^3a^	1.69 (1.19)	1.74 (1.13)	1.62 (1.26)	< 0.001
Quit in past year (cessation)^3^	9.2%	6.2%	12.6%	< 0.001
Tried to quit in past year (≥ 1 past-year quit attempt)^3^	33.9%	30.7%	37.5%	< 0.001
Number of past-year quit attempts^4b^	1.43 (1.79)	1.40 (1.77)	1.46 (1.79)	0.002
Use of cessation support^4^				
Prescription medication	7.1%	6.8%	7.4%	0.400
Behavioural support	7.4%	6.1%	8.7%	< 0.001
E-cigarettes	31.3%	32.7%	29.8%	0.029
Monthly inflation-adjusted national tobacco control expenditure (£)^c^	137,000 (247,000)	142,000 (237,000)	131,000 (261,000)	0.862

Data are shown as percentages or mean (SD), unless otherwise specified

There was a small amount of missing data for some variables (< 0.1% gender, 2.4% cigarette dependence, 4.1% tried to quit in the past year); valid percentages are shown

C2DE small employers/lower supervisory/technical/semi-routine/routine/never workers/long-term unemployed

^a^Strength of urges to smoke rated on a scale from 0 (none) to 5 (extremely strong)

^b^Geometric mean

^c^Population-level variable, unweighted

^1^Among 18–24-year-olds (*unweighted n* = *12,455*)

^2^Among 45–65-year-olds (*unweighted n* = *34,332*)

^3^Among past-year smokers (*unweighted n* = *17,964*)

^4^Among past-year smokers who tried to quit in the past year (*unweighted n* = *5754*)

^*^P value for the difference between samples recruited before vs. during pandemic. Chi-square tests for categorical variables and t-tests for continuous variables

### Current smoking

**Fig. 1:**
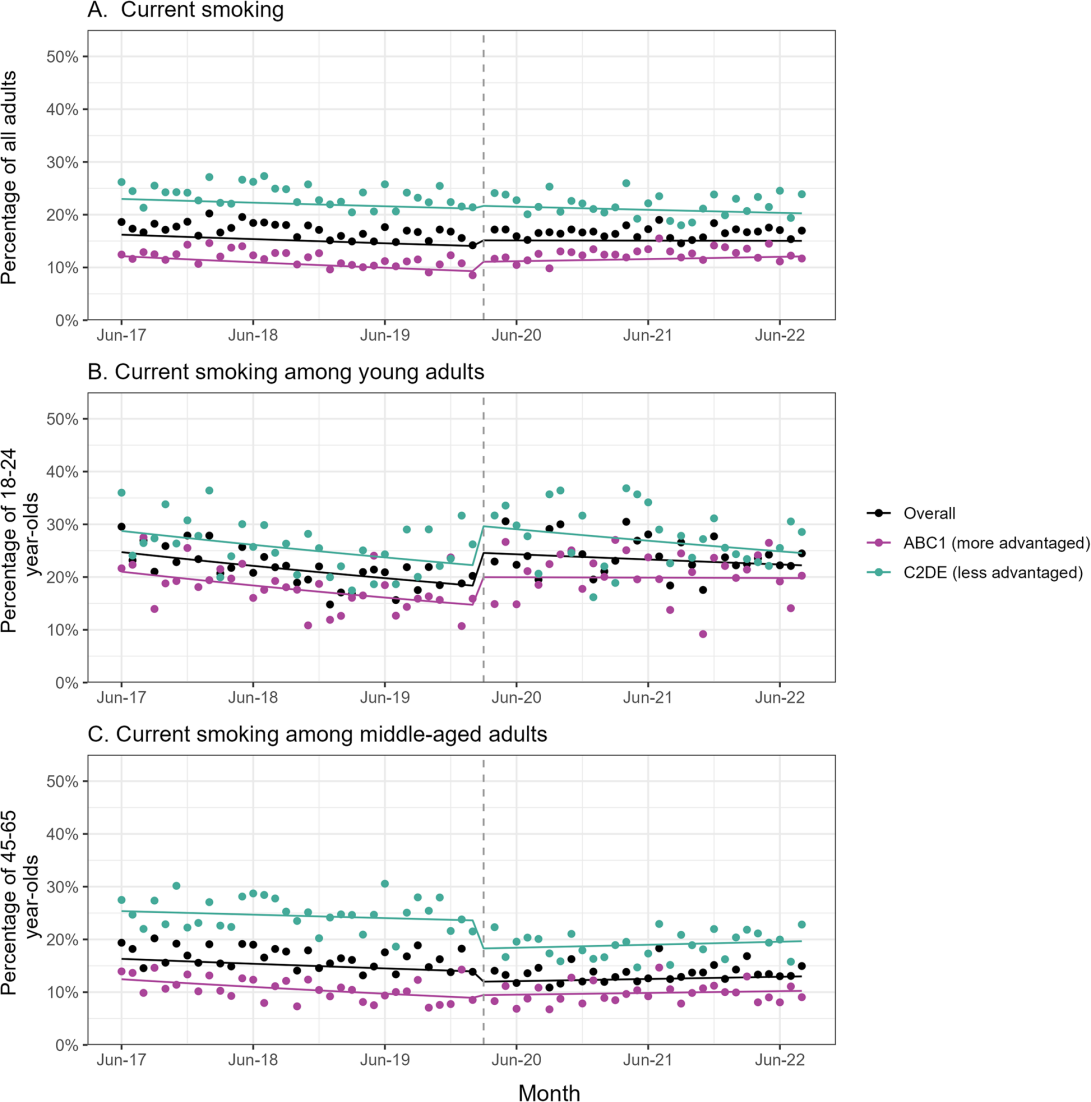
Current smoking, overall and by age and social grade. Panels show trends in the prevalence of current smoking among **A** adults in England (*unweighted n: overall* = *101,960, ABC1* = *64,088, C2DE* = *37,872*), **B** 18–24-year-olds (*unweighted n: overall* = *12,455, ABC1* = *7766, C2DE* = *4689*), and **C** 45–65-year-olds (*unweighted n: overall* = *34,332, ABC1* = *22,401, C2DE* = *11,931*), June 2017 to August 2022. Lines represent modelled weighted prevalence over the study period, adjusted for covariates. Points represent unadjusted weighted prevalence by month. The vertical dashed line indicates the timing of the start of the COVID-19 pandemic in England (March 2020). ABC1, managerial/professional/intermediate; C2DE, small employers/lower supervisory/technical/semi-routine/routine/never workers/long-term unemployed

Table 2 summarises the GAM results. Figure 1 shows trends in current smoking over the study period.

**Table 2:** GAM results: associations between the COVID-19 pandemic and smoking outcomes, overall and by social grade

	**Overall**			**Social grades ABC1** **(more advantaged)**			**Social grades C2DE** **(less advantaged)**		
	**% change**	**95% CI**		**% change**	**95% CI**		**% change**	**95% CI**	
		**Lower**	**Upper**		**Lower**	**Upper**		**Lower**	**Upper**
Current smoking^1a^									
Pre-pandemic trend (year*)	− 5.2	− 7.3	− 3.0	− 9.5	− 12.7	− 6.1	− 3.1	− 5.9	− 0.1
Pre vs. post step-level change	8.0	2.2	14.1	20.1	10.1	31.0	2.7	− 4.7	10.6
Pre vs. post Δ trend (year*)	5.2	1.4	9.0	14.5	8.3	21.1	0.3	− 4.5	5.4
Current smoking among young adults^2a^									
Pre-pandemic trend (year*)	− 10.5	− 15.4	− 5.4	− 12.4	− 19.1	− 5.2	− 9.1	− 16.2	− 1.4
Pre vs. post step-level change	34.9	17.7	54.7	36.9	12.7	66.3	34.2	10.1	63.6
Pre vs. post Δ trend (year*)	7.2	− 1.8	17.1	13.8	0.4	29.0	1.8	− 10.4	15.8
Current smoking among middle-aged adults^3a^									
Pre-pandemic trend (year*)	− 5.7	− 9.3	− 1.9	− 11.7	− 17.2	− 5.8	− 2.6	− 7.6	2.6
Pre vs. post step-level change	− 13.6	− 21.9	− 4.4	6.9	− 9.1	25.7	− 22.4	− 32.6	− 10.7
Pre vs. post Δ trend (year*)	9.6	2.5	17.2	17.1	5.5	30.0	5.8	− 3.6	16.2
Cessation^4b^									
Pre-pandemic trend (year*)	− 16.1	− 23.9	− 7.4	− 7.4	− 18.0	4.7	− 24.5	− 35.5	− 11.6
Pre vs. post step-level change	120.4	79.4	170.9	77.0	37.1	128.6	174.2	96.9	281.8
Pre vs. post Δ trend (year*)	21.9	7.9	37.9	3.2	− 11.8	20.7	45.4	20.0	76.2
Past-year quit attempt^4a^									
Pre-pandemic trend (year*)	− 8.2	− 11.7	− 4.5	− 6.7	− 11.7	− 1.5	− 9.0	− 13.8	− 3.8
Pre vs. post step-level change	41.7	29.7	54.7	34.9	19.3	52.7	46.0	29.0	65.4
Pre vs. post Δ trend (year*)	7.4	1.6	13.6	1.8	− 6.0	10.2	11.5	3.1	20.6
Number of past-year quit attempts^5a**^									
Pre-pandemic trend (year*)	3.0	0.3	5.8	4.3	0.6	8.1	2.0	− 1.9	6.1
Pre vs. post step-level change	− 3.0	− 8.9	3.2	− 10.7	− 17.9	− 2.9	3.4	− 5.6	13.3
Pre vs. post Δ trend (year*)	− 0.3	− 4.2	3.8	2.3	− 3.1	8.1	− 1.9	− 7.4	4.0
Use of prescription medication^5b^									
Time series trend (year*)	5.2	− 11.0	24.4	21.6	− 6.7	58.4	1.3	− 18.9	26.6
Pre vs. post step-level change	28.6	− 11.3	86.5	− 23.1	− 58.5	40.3	49.9	− 7.5	142.8
Pre vs. post Δ trend (year*)	− 15.5	− 33.8	8.0	− 14.0	− 42.0	27.5	− 17.4	− 39.9	13.5
Use of behavioural support^5b^									
Time series trend (year*)	− 16.2	− 30.4	0.9	− 21.0	− 39.2	2.6	− 12.4	− 33.0	14.3
Pre vs. post step-level change	133.0	55.3	249.6	120.4	21.2	300.8	136.3	34.2	316.1
Pre vs. post Δ trend (year*)	3.2	− 19.6	32.6	11.8	− 22.3	60.7	− 2.0	− 31.0	39.1
Use of e-cigarettes^5b^									
Time series trend (year*)	− 4.1	− 10.2	2.5	− 0.9	− 10.2	9.3	− 5.9	− 14.1	3.0
Pre vs. post step-level change	− 21.2	− 33.4	− 6.8	− 28.9	− 44.8	− 8.5	− 15.9	− 33.0	5.6
Pre vs. post Δ trend (year*)	23.2	11.1	36.5	24.1	6.1	45.2	22.5	6.7	40.8

*ABC1* managerial/professional/intermediate, *C2DE* small employers/lower supervisory/technical/semi-routine/routine/never workers/long-term unemployed, *CI* confidence interval

^a^Adjusted for seasonality, age, gender, social grade, and region

^b^Adjusted for seasonality, age, gender, social grade, region, cigarette dependence, and national expenditure on tobacco control mass media campaigns

^1^Among all adults

^2^Among 18–24-year-olds

^3^Among 45–65-year-olds

^4^Among past-year smokers

^5^Among past-year smokers who made ≥ 1 quit attempt in the past 12 months

^*^Monthly trends were analysed. We multiplied the coefficients by 12 to derive annual trends

^**^The number of quit attempts was analysed as a continuous variable and was log-transformed for analysis

Results are reported as percentage changes ((relative risk − 1)*100). Relative risks and associated 95% CIs are provided in Additional File 5: Table S2

Pre﻿ vs. post step-level change is the step-level change associated with the start of the pandemic. Pre vs. post Δ trend is the change in trend (slope) following the start of the pandemic (where the 95% CI does not overlap zero, this indicates trends differed significantly between the two time periods)

Overall, among adults in England, the onset of the COVID-19 pandemic was associated with a negligible step-level change in current smoking (Fig. 1A). However, there was a notable change in trend. Before the pandemic, smoking prevalence fell by 5.2% per year (relative risk, trend [RR_trend_] = 0.948; note this percentage represents the relative rather than absolute percentage point reduction, i.e. a 5.2% decrease compared to the previous year [(1-RR)*100], rather than a decrease of 5.2 percentage points within a given year). After the onset of the pandemic, this rate of decline slowed to 0.3% per year (RR_trend_ × RR_Δtrend_ = 0.948 × 1.052 = 0.997; Fig. 1A). The change in trend from pre- to post-onset of the pandemic was significant (relative risk, change in trend [RR_Δtrend_] = 1.052, 95% confidence interval [CI] = 1.014,1.090). In June 2017, smoking prevalence was estimated at 16.2%. At the start of the pandemic (March 2020), it was 15.1%. In August 2022, it was virtually unchanged, at 15.0%.

Stratified analyses showed a 20.1% (95% CI = 10.1, 31.0%) step-level increase in smoking prevalence among adults from more advantaged social grades (ABC1) at the start of the pandemic, followed by a slowing in the pre-pandemic decline to the point where progress in reducing smoking reversed (+ 3.6% per year compared with − 9.5% per year before the pandemic, RR_Δtrend_ = 1.145, 95% CI = 1.083,1.211; Fig. 1A). By contrast, there was no increase in smoking prevalence among those from less advantaged social grades (C2DE), and it appeared the modest (~ 3% per year) pre-pandemic decline continued (Fig. 1A).

When we looked at current smoking in different age groups, we saw divergent changes associated with the pandemic: a 34.9% (95% CI = 17.7,54.7%) step-level increase among 18–24-year-olds (Fig. 1B) but a 13.6% (95% CI = 4.4, 21.9%) step-level decrease among 45–65-year-olds (Fig. 1C). While the rise in smoking among young adults was similar across social grades, the fall among middle-aged adults was limited to those from less advantaged social grades (− 22.4%, 95% CI =  − 10.7, − 32.6%). As we observed overall, progress in reducing smoking stopped among more advantaged social grades during the pandemic (from − 12.4% to − 0.3% per year among 18–24-year-olds, RR_Δtrend_ = 1.138, 95% CI = 1.004, 1.290; and from − 11.7% to + 3.4% per year among 45–65-year-olds, RR_Δtrend_ = 1.171, 95% CI = 1.055–1.300) but was similar to pre-pandemic rates within less advantaged social grades (Fig. 1B and C).

The data indicated these changes were sustained over time (Fig. 1), rather than short-lived pulse effects during the early months of the pandemic (Additional File 5: Table S3).

### Quitting activity

**Fig. 2:**
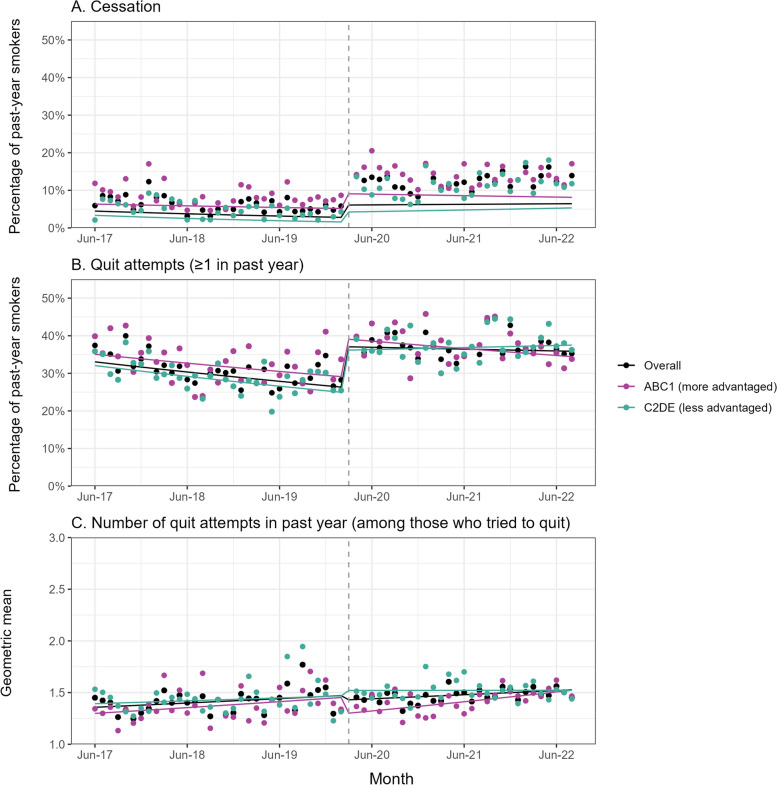
Quitting activity, overall and by social grade. Panels show trends in the prevalence of **A**) cessation and **B** making at least one quit attempt in the past year among past-year smokers (*unweighted n: overall* = *17,964, ABC1* = *8802, C2DE* = *9162*), and **C** the weighted geometric mean number of past-year quit attempts among past-year smokers who made at least one quit attempt (*unweighted n: overall* = *5754, ABC1* = *2908, C2DE* = *2846*), June 2017 to August 2022. Lines represent modelled weighted prevalence (or means) over the study period, adjusted for covariates. Points represent unadjusted weighted prevalence (or means) by month. The vertical dashed line indicates the timing of the start of the COVID-19 pandemic in England (March 2020). Corresponding data without adjustment for dependence are shown in Additional File 5: Fig. 1 and Additional File 5: Table 4. ABC1, managerial/professional/intermediate; C2DE, small employers/lower supervisory/technical/semi-routine/routine/never workers/long-term unemployed

Data on cessation were available for all of the 17,964 past-year smokers in our sample. There were 741 (4.1%) with missing data on quit attempts and, among those eligible, 0 with missing data on the number of quit attempts. Table 2 summarises the GAM results. Figure 2 shows trends in quitting activity over the study period.

Among past-year smokers, the pandemic was associated with a 120.4% (95% CI = 79.4–170.9%) step-level increase in cessation (Fig. 2A). This increase was similar at 154.4% (95% CI = 104.8–216.1%) when cigarette dependence was not adjusted for (Additional File 5: Table S4; Additional File 5: Fig. S1A) despite mean cigarette dependence only decreasing very slightly during the pandemic (Additional File 5: Table S5; Additional File 5: Fig. S3). There was also a change in trend: the prevalence of cessation was reducing before the pandemic at a rate of 16.1% per year (RR_trend_ = 0.839); this rate of decline slowed during the pandemic (RR_Δtrend_ = 1.219, 95% CI = 1.079–1.379) to 2.3% (Fig. 2A). The change in trend was driven by the less advantaged social grades, among whom the rate of cessation was reversed from − 24.5% per year before the pandemic to + 9.8% per year during the pandemic (RR_Δtrend_ = 1.454, 95% CI = 1.200–1.762; Fig. 2A). By contrast, the more modest (7.4%) pre-pandemic decline in cessation among those from more advantaged social grades appeared to continue (Fig. 2A). This pattern of results was largely replicated when we analysed data separately for smokers aged ≥ 25 years (Additional File 5: Table S6; Additional File 5: Fig. S4). However, among the much smaller group aged 18–24 years, while we observed a significant step-level increase in cessation, there was uncertainty in all the other results with the confidence intervals crossing zero and including the point estimate from the overall analyses for the trend in cessation before the pandemic, the change in trend, and the patterning of the socio-economic results (Additional File 5: Table S6; Additional File 5: Fig. S4).

The pandemic was also associated with a 41.7% (95% CI = 29.7–54.7%) step-level increase in the proportion of past-year smokers who made ≥ 1 quit attempt (Fig. 2B). This increase occurred across ages but was larger among smokers aged 18–24 (90.8% [95% CI = 57.0–131.9%]) than those aged ≥ 25 (31.5% [95% CI = 19.1–45.2%]) (Additional File 5: Table S6; Additional File 5: Fig. S4). The rate of decline in quit attempts slowed from 8.2 to 1.4% per year (RR_Δtrend_ = 1.074, 95% CI = 1.016–1.136; Fig. 2B); again, this was driven by those from less advantaged social grades, with no significant change in trend among the more advantaged social grades (Fig. 2B), and was only observed among those aged ≥ 25 (Additional File 5: Table S6; Additional File 5: Fig. S4). Among those who tried to quit, there was little change in the mean number of attempts made (Fig. 2C).

While analyses of pulse effects showed increases in quitting activity in the first 2–3 months of the pandemic (Additional File 5: Table S3), it is clear from visual inspection of the data in Fig. 2 and the change in trend results (Table 2) that these increases were sustained through to August 2022.

### Use of cessation support

**Fig. 3:**
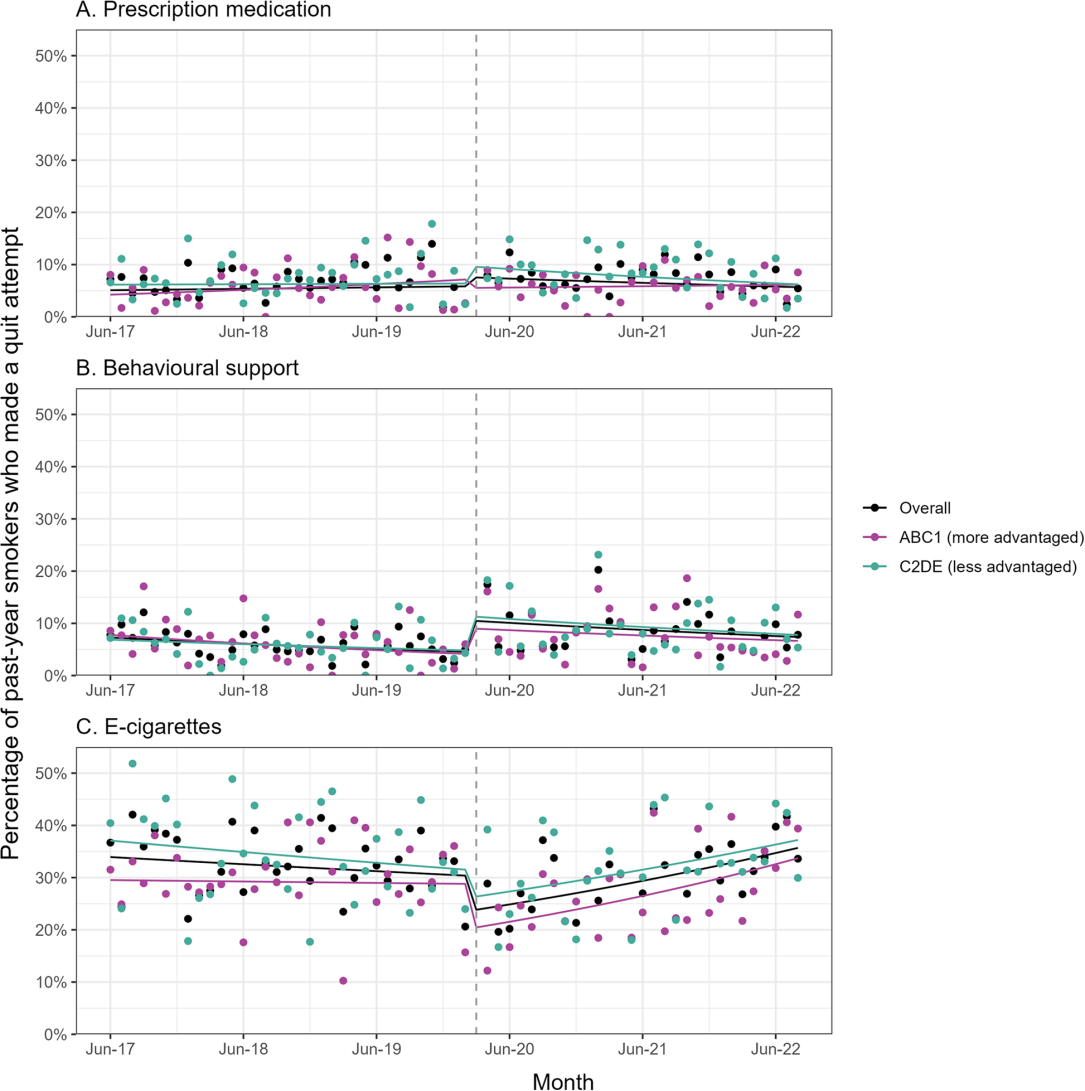
Use of support by smokers in quit attempts, overall and by social grade. Panels show trends in the prevalence of use of **A** prescription medication, **B** behavioural support, and **C** e-cigarettes in the most recent quit attempt among past-year smokers who made a least one quit attempt (*unweighted n: overall* = *5754, ABC1* = *2908, C2DE* = *2846*), June 2017 to August 2022. Lines represent modelled weighted prevalence over the study period, adjusted for covariates. Points represent unadjusted weighted prevalence by month. The vertical dashed line indicates the timing of the start of the COVID-19 pandemic in England (March 2020). Corresponding data without adjustment for dependence are shown in Additional File 5: Fig. 2 and Additional File 5: Table 4. ABC1, managerial/professional/intermediate; C2DE, small employers/lower supervisory/technical/semi-routine/routine/never workers/long-term unemployed

Table 2 summarises the GAM results. Figure 3 shows trends in use of cessation support over the study period.

Among past-year smokers who made a quit attempt, the onset of the COVID-19 pandemic was associated with little change in the use of prescription medication (Fig. 3A). Point estimates for a step-level change were in opposite directions for those from more and less advantaged social grades, but neither group had a statistically significant change. This finding was robust to the exclusion of varenicline from this variable (Additional File 5: Table 7).

However, the pandemic was associated with changes in the use of behavioural support and e-cigarettes for quitting smoking. There was a 133.0% (95% CI = 55.3–249.6%) step-level increase in use of behavioural support, followed by a continuation of the modest pre-pandemic decline (Fig. 3B). By contrast, there was a 21.2% (95% CI = 6.8–33.4%) step-level decrease in use of e-cigarettes (Fig. 3C). This change was short-lived (Additional File 5: Table 3) because there was also a change in trend, reversing this step-level decline: before the pandemic, the proportion of smokers using e-cigarettes in a quit attempt fell by 4.1% per year; during the pandemic, it increased by 18.1% per year (RR_Δtrend_ = 1.232, 95% CI = 1.111–1.365, Fig. 3C). These changes were similar across social grades.

Changes in the use of cessation support were similar when cigarette dependence was not adjusted for (Additional File 5: Table 4; Additional File 5: Fig. 2).

## Discussion

Before the COVID-19 pandemic, smoking prevalence had been falling among adults in England at a near linear rate for more than 20 years [25]. Our data show that this decline almost completely stopped since the pandemic began, resulting from changes in smoking and quitting behaviours. There were sustained changes in smoking prevalence in different age groups: a step increase among 18–24-year-olds, indicating a potential rise in the uptake of smoking, offset by a step decrease among 45–65-year-olds, which also suggested no evidence of a substantial rise in late relapse. In both age groups, these step-level changes were followed by the pre-pandemic declines stopping, and prevalence remaining flat. Inequalities in smoking prevalence appear to have narrowed, but for the wrong reasons: while the pre-pandemic trend did not change among less advantaged social grades, the pre-pandemic decline halted among the more advantaged. There were sustained increases in quitting activity among past-year smokers: cessation rates (i.e. the proportion who reported having stopped smoking in the past year) more than doubled during the pandemic, and there was also an increase in the rate of past-year quit attempts. Among smokers from more disadvantaged social grades, declining pre-pandemic trends in cessation and quit attempts flattened or reversed. Among those who tried to quit, there was little change in the number of attempts made or in the use of prescription medication in a quit attempt, but there was a short-term rise in the use of behavioural support and a short-term fall in the use of e-cigarettes, which recovered during the pandemic period.

These results build upon and extend our previous analysis of changes in smoking and quitting during the first COVID-19 lockdown in England (April–July 2020) [1]. We applied more complex modelling techniques to data collected over a longer period (up to August 2022), offering insight into the longer-term impact of the pandemic on smoking. In addition, we analysed additional outcomes (e.g. relapse) to provide a more complete picture of the pandemic’s impacts. There are three key findings.

First, the pandemic has been associated with opposing changes in smoking behaviour: the proportion of smokers who have tried to quit and succeeded has risen, but so has the proportion of young adults who smoke. In the absence of any adverse effect on relapse, the net result is stable overall smoking prevalence, which appears to have halted years of steady decline in prevalence pre-pandemic. As we have discussed previously [11], the unique circumstances brought about by the pandemic may have prompted some smokers to quit (e.g. by providing a ‘teachable moment’, disrupting daily routines, reducing social smoking cues, or onset of new chronic health conditions caused by the pandemic, especially among older adults) while encouraging other people to start smoking (e.g. to relieve stress or boredom). In particular, younger adults have experienced higher levels of stress, upheaval, and social isolation during the pandemic [26, 27], which might have contributed to increased smoking prevalence in this group. Based on the stagnation in the decline of smoking prevalence in the two years since the pandemic began, there is an urgent need for bold policy action. Even based on pre-pandemic trends, the UK Government’s smokefree 2030 target would not have been achieved until at least 2037 [28]. The stagnation appears to have been caused by an increase in smoking among young adults. There is a need to understand the motives driving young adults to take up smoking to develop interventions and public health messaging to combat this. Increasing the age of sale of cigarettes is one strategy that may be effective both in reducing uptake and in narrowing inequalities in smoking [29, 30]. It is also important to keep momentum going in terms of increased quitting activity. Investment in national tobacco control mass media activity may be an efficient strategy, given substantial evidence linking such campaigns to increased rates of success in stopping smoking [16, 17, 31, 32].

Second, the pandemic appears to have had equity-positive impacts on smoking. Progress in reducing smoking prevalence has historically been slower for disadvantaged groups [33, 34]. However, while the (considerable) pre-pandemic decline in smoking prevalence among the more advantaged social grades levelled off during the pandemic, the more modest decline among the less advantaged social grades continued. While it is encouraging to see inequalities in smoking narrow during the pandemic, ideally this would be the result of accelerating reductions in smoking prevalence among disadvantaged groups, rather than slowing the decline among advantaged groups. Trends in quitting activity also showed evidence of an apparent narrowing of inequalities: a consistent decline among those from more advantaged social grades but a levelling off during the pandemic among less advantaged social grades. Possible explanations for these differences include those from less advantaged social grades being more likely to experience financial impacts of the pandemic (e.g. due to job loss or reduced earnings) which make (taking up or continuing) smoking less affordable, or work in front-line jobs that increase exposure to COVID-19 and might make quitting smoking higher priority [35–37]. In addition, manual jobs were less disrupted through the pandemic, whereas many non-manual jobs switched to home working, leading to loneliness and poorer mental health [38, 39], which may have made people in more advantaged social grades less inclined to try to stop smoking. In working toward the smokefree 2030 target, there is a need for action to reignite progress in reducing smoking among the more advantaged social grades and identify ways to accelerate the decline among less advantaged groups.

Finally, the pandemic has not had an enduring impact on the use of evidence-based support by smokers trying to quit. At the start of the pandemic, there was a fall in the use of e-cigarettes, the most popular quitting aid used by smokers in England [24]. It is possible this resulted from concerns that vaping might exacerbate the risk of contracting or experiencing complications from COVID-19 [40], or difficulties in getting to vape shops before businesses pivoted, which were not exempted from lockdown rules. The decline in e-cigarette use was offset by a rise in the use of behavioural support (e.g. stop smoking services or digital support via websites or apps), so there was no adverse impact on the success of quit attempts (as indicated by an increased rate of cessation). Given stop smoking services were not able to provide in-person support when lockdown restrictions were introduced, it is likely that the use of other forms of behaviour support included in our measure that could be accessed remotely (e.g. websites and apps) may have driven the short-term increase we observed (although stop smoking services were rapidly reconfigured to provide remote support via telephone and video calls [41]). Over the longer term, the use of different types of support returned to pre-pandemic levels. Given most smokers who tried to quit during the pandemic did not report using any evidence-based support, there remains a substantial opportunity to boost success in quitting by directing smokers to effective support. Increased investment in national tobacco control mass media campaigns may be a cost-effective means to achieve this [16, 31].

Strengths of this study include the large, representative sample, the repeat cross-sectional design with data pre-dating the pandemic, and the broad range of data captured on smoking and quitting behaviour. There were also limitations. First, we used a hybrid sampling approach rather than random probability sampling. However, comparisons with other sources suggest the survey recruits a nationally representative sample and produces similar estimates of key smoking variables [9, 10]. Second, there is no direct assessment of late relapse in the Smoking Toolkit Study. We therefore analysed changes in smoking prevalence among 45–65-year-olds as a proxy variable on the basis that any increase in this age group would most likely be driven by a rise in late relapse rather than uptake. However, it is possible that any small increase in late relapse among this group was offset by more people quitting. Third, the modality of data collection changed from face-to-face (before the pandemic) to telephone interviews (during the pandemic). While this was unavoidable due to social distancing restrictions, it is possible that it contributed to some of the changes we observed — especially step-level changes, which would be most sensitive to any effects of the switch. Step-level changes may also be an artefact of the models used (e.g. if there is a slight curvature in the association, the fitted lines can deviate from the data at either end of the range of the predictor, and the lack of fit can give the impression of a genuine step-level change), so should be interpreted cautiously. Nevertheless, comparisons of the face-to-face and telephone data within the Smoking Toolkit Study [12], combined with previous studies showing a high degree of comparability between face-to-face and telephone interviews [42, 43], suggest that it is reasonable to compare data collected via the two methods. Given there were no further updates to methods after April 2020, changes in the slope of trends are unlikely to be explained by the switch in methodology. Fourth, outcomes related to cessation were retrospectively reported, introducing scope for recall bias. This may have particularly affected the number of quit attempts and the duration of abstinence. However, our definition of cessation relied on current abstinence at the time of the survey and should therefore not have been affected by inaccurate recall. Finally, quitting outcomes were assessed in the context of the last 12 months. This is because we did not have a sufficient sample size to undertake a meaningful analysis of (rarer) shorter-term quitting outcomes. The time frame for these outcomes may have caused us to underestimate step-level changes, because any effects of the pandemic onset will have been diluted by the outcomes including data from before the pandemic. It is also possible that it may not have affected those surveyed before and since the pandemic equally if, for instance, reported quit attempts were less versus more recent from before to after the pandemic started.

## Conclusions

Reductions in smoking prevalence among middle-aged adults and sustained increases in quit attempts and cessation among smokers during the COVID-19 pandemic have been offset by a sustained rise in uptake among young adults. As a result, the rate of decline in adult smoking prevalence in England has stagnated. Changes in use of support predominantly occurred in the early stages of the pandemic and have since returned to usual levels. There was no evidence to suggest the pandemic increased the risk of early or late relapse. The slowing in the rate of decline in smoking prevalence was pronounced in more advantaged social grades.

### Supplementary Information

**Additional file 1. ** Literature review.

**Additional file 2. ** Study protocol.

**Additional file 3. ** Checklist.

**Additional file 4. ** Measures.

**Additional file 5: Table S1-7, Figs S1-4.** Table S1 – Unweighted descriptive statistics. **Table S2** – GAM results (relative risks). **Table S3** – Sensitivity analysis: pulse effects. **Table S4** – Sensitivity analysis: excluding dependence. **Fig S1** – Quitting activity, with and without adjustment for dependence. **Fig S2** – Use of support, with and without adjustment for dependence. **Table S5** – Associations with cigarette dependence. **Fig S3** – Cigarette dependence. **Table S6** – Sensitivity analysis: cessation and quit attempts by age. **Fig S4** – Cessation and quit attempts by age and social grade. **Table S7** – Sensitivity analysis: excluding varenicline.

## Data Availability

Data and code are available from the corresponding author on reasonable request.

## Abbreviations

CI: Confidence interval
GAM: Generalised additive model
RR_trend_: Relative risk, trend
RR_Δtrend_: Relative risk, change in trend
UCL: University College London
