# Supplementary material for: Have there been sustained impacts of the COVID-19 pandemic on trends in smoking prevalence, uptake, quitting, use of treatment, and relapse? A monthly population study in England, 2017–2022
Source: BMC Med. 2023 Dec 14;21:474. doi: 10.1186/s12916-023-03157-2 (PMC10720231; doi:10.1186/s12916-023-03157-2)
Supplement: Supplementary file 1 — Additional file 1. Literature review. [file 12916_2023_3157_MOESM1_ESM.pdf]

The extent to which the Covid-19 pandemic has affected ***smoking prevalence*** remains unclear. A meta-analysis of international studies up to November 2020 reporting on changes in smoking behaviour after the onset of the pandemic indicated there had been a significant decline in smoking prevalence (12 studies; prevalence ratio=0.87, 95%CI [confidence interval]=0.79-0.97) [2]. However, there was a very high degree of heterogeneity across studies (I^2=99.3%), with some reporting a significant increase in prevalence, some a significant decrease, and others no change. In addition, just two studies were representative, with the majority using convenience samples recruited via social media or other online platforms, and almost all were judged to be at high risk of bias. Further data on changes in smoking prevalence were published in 2021. In the UK, the Annual Population Survey (a large, representative household survey) showed a large but implausible decline in the proportion of adults who smoked cigarettes, from 13.8% in the first quarter of 2020 to 12.3% in April to December of 2020. The magnitude of the decline may have been partially attributable to the change in mode of data collection from predominantly face-to-face interviews to telephone-only [5]. The Opinions and Lifestyle Survey, another representative survey which was already remote at the start of the pandemic (telephone/online), suggested smoking prevalence increased at the start of the pandemic, from 15.3% in April 2020 to a peak of 16.3% in August 2020 (although this change was not statistically significant), before falling significantly to 12.5% in December 2020 [5]. The Smoking Toolkit Study (a representative monthly survey) showed no overall change in smoking prevalence among adults in England during the first Covid-19 lockdown in England (despite a change from face-to-face to telephone interviews), but detected a large increase in smoking prevalence among young adults (18-34 years; from 21.5% between August 2019 and February 2020 to 26.8% between April and July 2020) [1].

Data on changes in ***uptake*** of smoking during the Covid-19 pandemic are scarce. Sarich et al.'s meta-analysis [2] identified four studies that provided data on the proportion of smokers who started or restarted smoking during the pandemic; the pooled estimate was 2%, with a high degree of heterogeneity between studies (I^2=91.7%). All the studies used convenience sampling and were judged at high risk of bias. A survey of young people in Great Britain by Action on Smoking and Health showed no evidence of an increase in uptake (indexed by ever-use of cigarettes) among 11-17 year-olds [6]. Analysing changes in smoking prevalence among young adults could offer insight into any impact of the pandemic on uptake, as increases in prevalence in this age group would likely be largely driven by uptake rather than relapse.

More data are available on ***quitting***, with studies indicating the pandemic may have prompted many smokers to stop. The Smoking Toolkit Study showed substantial increases in the overall rate of cessation among smokers (from 3.9% in August 2019 to February 2020 to 10.0% in April to July 2020) and the success rate of quit attempts (from 12.7% to 25.3%) [1]. The proportion of younger smokers (18-34 years) who reported making a serious attempt to quit increased by 39.9% (from 32.1% to 44.9%) [1]. The ITC Four Country survey conducted in April-June 2020 found almost half (46.7%) of current smokers had thought about quitting because of Covid-19 [3]. Roughly equal proportions reported having reduced (14.2%) or increased (14.6%) the amount they smoked since the coronavirus outbreak. Smokers who reported positive behaviour change (quit attempt/reduction) tended to be less dependent, more concerned about personal susceptibility to infection, and were more likely to believe that Covid-19 is more severe for smokers. There was substantial between-country variation: smokers in Australia were less likely than those in England, Canada, or the United States to have tried to quit or reduced their smoking, which may be attributable to the significantly

lower impact of Covid-19 on Australia during the early stages of the pandemic. It is not clear whether the pandemic has had an influence on the frequency of quit attempts.

There is limited evidence on changes in ***use of cessation support*** since the start of the pandemic among smokers who have tried to quit. The Smoking Toolkit Study showed an increase in the proportion of smokers making a quit attempt using remote support (telephone support/websites/apps; from 2.4% in August 2019 to February 2020 to 6.8% in April to July 2020) and a decline in the proportion using evidence-based support (defined as face-to-face behavioural support, prescription medication [varenicline, bupropion, or nicotine replacement therapy (NRT)], e-cigarettes, or over-the-counter NRT; from 53.8% to 44.8%), but these changes were not statistically significant over and above changes over the same period a year earlier [1]. These analyses were limited by the small number of smokers reporting a past-year quit attempt [1], so cannot be considered conclusive.

Finally, relatively little is known about what impact the pandemic has had on ***relapse*** to smoking among ex-smokers. Given studies have generally observed large increases in quitting activity [1,3] but little or no reduction in smoking prevalence [1-2,5], it is possible that increased rates of cessation are being offset by increased rates of relapse. Examining changes in smoking prevalence among middle-aged and older adults could offer insight into the impact of the pandemic on late relapse, as any increases in smoking prevalence in this age group would likely be largely driven by relapse rather than uptake (as few people take up smoking after the age of 25 [7]).
