# Supplementary material for: Have there been sustained impacts of the COVID-19 pandemic on trends in smoking prevalence, uptake, quitting, use of treatment, and relapse? A monthly population study in England, 2017–2022
Source: BMC Med. 2023 Dec 14;21:474. doi: 10.1186/s12916-023-03157-2 (PMC10720231; doi:10.1186/s12916-023-03157-2)
Supplement: Supplementary file 2 — Additional file 2. Study protocol. [file 12916_2023_3157_MOESM2_ESM.pdf]

UCL Tobacco and Alcohol Research Group

Study Protocol

Version 7
11 October 2022

**Title**	Have there been sustained impacts of the Covid-19 pandemic on trends in smoking prevalence, uptake, quitting, use of treatment, and relapse? A monthly population study in England, 2017-2022
**Authors**	Sarah Jackson, Emma Beard, Harry Tattan-Birch, Lion Shahab, Jamie Brown<br><br>1 Department of Behavioural Science and Health, University College London, UK<br>2 SPECTRUM Consortium, UK
**Rationale**	The Covid-19 pandemic has had a profound impact on everyday life, public health, and health services. The emergence of dangerous variants suggests there may be continuing cyclical consequences for several years (1,2). Studies conducted during the early stages of the pandemic documented mixed changes in smoking behaviour, including evidence of both increased and decreased smoking prevalence and higher rates of quit attempts and cessation among smokers (e.g., 3–5). Identifying sustained changes in smoking prevalence, uptake, quitting, use of treatments, and relapse following the onset of the Covid-19 pandemic, and the groups in which they have occurred, is essential for building a clear picture of its public health impact and targeting policy, messaging, and support services.<br><br>The extent to which the Covid-19 pandemic has affected smoking prevalence remains unclear. A meta-analysis of international studies up to November 2020 reporting on changes in smoking behaviour after the onset of the pandemic indicated there had been a significant decline in smoking prevalence (12 studies; prevalence ratio 0.87, 95% CI 0.79-0.97) (4). However, there was a very high degree of heterogeneity across studies (I^2 99.3%), with some reporting a significant increase in prevalence, some a significant decrease, and others no change. In addition, just two studies were representative, with the majority using convenience samples recruited via social media or other online platforms, and almost all were judged to be at high risk of bias. Further data on changes in smoking prevalence were published in 2021. In the UK, the Annual Population Survey (a large, representative household survey) showed a large but implausible decline in the proportion of adults who smoked cigarettes, from 13.8% in the first quarter of 2020 to 12.3% in April to December of 2020. The magnitude of the decline may have been partially attributable to the change in mode of data collection from predominantly face-to-face interviews to telephone-only (6). The Opinions and Lifestyle Survey, another representative survey which was already remote at the start of the pandemic (telephone/online), suggested smoking prevalence increased at the start of the pandemic, from 15.3% in April 2020 to a peak of 16.3% in August 2020 (although this change was not statistically significant), before falling significantly to 12.5% in December 2020 (6). The Smoking Toolkit Study (a representative monthly survey) showed no overall change in smoking prevalence among adults in England during the first Covid-19 lockdown in England (despite a change from face-to-face to telephone interviews), but detected a large increase in smoking prevalence among young adults (18-34 years; from 21.5% between August 2019 and February 2020 to 26.8% between April and July 2020) (3).

	Data on changes in uptake of smoking during the Covid-19 pandemic are scarce. Sarich et al.'s meta-analysis (4) identified four studies that provided data on the proportion of smokers who started or restarted smoking during the pandemic; the pooled estimate was 2%, with a high degree of heterogeneity between studies (I^2 91.7%). All the studies used convenience sampling and were judged at high risk of bias. A survey of young people in Great Britain by Action on Smoking and Health (ASH) showed no evidence of an increase in uptake (indexed by ever use of cigarettes) among 11-17 year-olds (7). Analysing changes in smoking prevalence among young adults could offer insight into any impact of the pandemic on uptake, as increases in smoking prevalence in this age group would likely be largely driven by uptake rather than relapse.<br><br>More data are available on quitting, with studies indicating the Covid-19 pandemic may have prompted many smokers to stop. The Smoking Toolkit Study showed substantial increases in the overall rate of cessation among smokers (from 3.9% in August 2019 to February 2020 to 10.0% in April to July 2020) and the success rate of quit attempts (from 12.7% to 25.3%) (3). In addition, the proportion of younger smokers (18-34 years) who reported making a serious attempt to quit increased by 39.9% (from 32.1% to 44.9%) (3). The ITC Four Country survey conducted in April-June 2020 found almost half (46.7%) of current smokers had thought about quitting because of Covid-19 (5). Roughly equal proportions reported having reduced (14.2%) or increased (14.6%) the amount they smoked since the coronavirus outbreak. Smokers who reported positive behaviour change (quit attempt/reduction) tended to be less dependent, more concerned about personal susceptibility to infection, and were more likely to believe that Covid-19 is more severe for smokers. There was substantial between-country variation: smokers in Australia were less likely than those in England, Canada, or the United States to have tried to quit or reduced their smoking, which may be attributable to the significantly lower impact of Covid-19 on Australia during the early stages of the pandemic. It is not clear whether the pandemic has had an influence on the frequency of quit attempts.<br><br>There is limited evidence on changes in use of cessation support since the start of the pandemic among smokers who have tried to quit. The Smoking Toolkit Study showed an increase in the proportion of smokers who made a quit attempt using remote support (telephone support, websites, and apps; from 2.4% in August 2019 to February 2020 to 6.8% in April to July 2020) and a decline in the proportion using evidence-based support (defined as face-to-face behavioural support, prescription medication [varenicline, bupropion, or nicotine replacement therapy (NRT)], e-cigarettes, or NRT bought over the counter; from 53.8% to 44.8%), but these changes were not statistically significant over and above changes over the same period a year earlier (3). These analyses were limited by the small number of smokers reporting a past-year quit attempt (3), so cannot be considered conclusive.<br><br>Finally, relatively little is known about what impact the pandemic has had on relapse to smoking among ex-smokers. Given studies have generally observed large increases in quitting activity (3,5) but little or no reduction in smoking prevalence (3,4,6), it is possible that increased rates of cessation are being offset by increased rates of relapse. Examining changes in smoking prevalence among middle-aged and older adults could offer insight into the impact of the pandemic on late relapse, as any increases in smoking prevalence in this age group would likely be largely driven by relapse rather than uptake (as few people take up smoking after the age of 25 (8)).<br><br>It is not yet clear whether the short-term changes in smoking patterns reported in these studies have translated into long-term, sustained changes. As highlighted by

	Sarich et al. (4), there is some evidence that lifestyle behaviours adopted during a pandemic can persist for some time – for example, sustained increases in alcohol abuse/dependence symptoms were observed three years after the 2003 SARS outbreak among individuals in China who were quarantined or worked in high-risk settings during the epidemic (9). On the other hand, it is possible that once life started to return to 'normal' after the early months of the pandemic, people reverted to their previous smoking patterns and quitting became a less salient issue.
**Aim and research questions**	Two and a half years on from the start of the pandemic, there are now sufficient data within the Smoking Toolkit Study to undertake a more detailed analysis of whether there has been a sustained impact of the Covid-19 pandemic era on smoking and smoking cessation patterns in England. In collecting data monthly, the Smoking Toolkit Study affords a unique opportunity to assess detailed trends at this stage (most representative surveys collect these data at much less frequent intervals).<br><br>Specifically, we aim to address the following research questions. Using data from June 2017 through August 2022:<br>1. What has been the sustained impact of the Covid-19 pandemic on monthly trends in:<br>a. Current smoking (among all adults);<br>b. Cessation and making ≥1 serious quit attempt (among past-year smokers);<br>c. Number of past-year quit attempts and use of cessation support in the most recent attempt (among past-year smokers who made ≥1 quit attempt);<br>d. Uptake of smoking (indexed by current smoking among young adults);<br>e. Short-term relapse (indexed by failure of quit attempts that started 6-12 months before the survey);<br>f. Late relapse (indexed by current smoking among middle-aged adults)?<br>We will also explore the impact of the Covid-19 pandemic on these outcomes separately by socioeconomic position.
**Methods**	
**Design**	Data will be drawn from the ongoing Smoking Toolkit Study, a monthly cross-sectional survey of a representative sample of adults in England designed to provide insights into population-wide influences on smoking and cessation by monitoring trends on a range of variables relating to smoking (10). The study uses a hybrid of random probability and simple quota sampling to select a new sample of approximately 1,700 adults aged ≥16 years each month. England is split into more than 170,000 output areas (consisting of approximately 300 households each). These output areas are stratified by Acorn characteristics (an established geo-demographic analysis of the population; http://www.caci.co.uk/acorn/ and geographic region then randomly selected to be included in an interviewer's list. Interviews are performed with one household member until quotas based on factors influencing the probability of being at home (e.g. gender, age, working status) are fulfilled. Comparisons with sales data and other national surveys indicate that key variables such as key sociodemographic variables, smoking prevalence, and cigarette consumption are nationally representative (10,11).<br><br>Data were collected monthly through face-to-face computer-assisted interviews. However, social distancing restrictions under the Covid-19 pandemic meant that no data were collected in March 2020 and data from April 2020 onwards were collected via telephone, and the lower age bound for participation was increased from 16 to 18 years. The telephone-based data collection relied upon the same combination of

	random location and quota sampling, and weighting approach as the face-to-face interviews and comparisons of the two data collection modalities indicate good comparability (3,12,13).<br><br>For the present study, we will use individual-level data from respondents to the survey in the period from June 2017 through August 2022. We selected June 2017 as the first month of data for this analysis because it provides a period with a relatively stable tobacco control climate in England (following implementation of the Tobacco Products Directive between May 2016 and May 2017) meaning effects of the pandemic on smoking outcomes can more easily be detected. Because the sample was restricted to people aged ≥18 years when data collection switched from face-to-face to telephone interviews, we will exclude any participants aged 16–17 recruited before April 2020 for consistency.
**Measures**	*Outcome variables*<br><br>We will assess the following smoking outcomes:<br>• Among all adults: current smoking;<br>• Among past-year smokers: cessation, quit attempts;<br>• Among past-year smokers who made ≥1 quit attempt in the past 12 months: number of quit attempts made, use of prescription medication, use of behavioural support, use of e-cigarettes;<br>• Among 18-24 year-olds: uptake of smoking (indexed by current smoking);<br>• Among past-year smokers who made ≥1 quit attempt 6-12 months before completing the survey: short-term relapse to smoking;<br>• Among 45-65 year-olds: late relapse (indexed by current smoking).<br><br>Smoking status will be assessed with the question: 'Which of the following best applies to you? (a) I smoke cigarettes (including hand-rolled) every day; (b) I smoke cigarettes (including hand-rolled), but not every day; (c) I do not smoke cigarettes at all, but I do smoke tobacco of some kind (e.g. pipe, cigar or shisha); (d) I have stopped smoking completely in the last year; (e) I stopped smoking completely more than a year ago; (f) I have never been a smoker (i.e. smoked for a year or more)'. ***Current smoking*** will be coded 1 for those who report smoking any type of tobacco [i.e. responses (a–c)] and 0 for those who report being a former or never smoker [responses (d–f)]. Past-year smoking will be coded 1 for those who report current smoking or having stopped in the past year [responses (a–d)] and 0 for those who report stopping more than a year ago or never smoking [responses (e–f)].<br><br>Among past-year smokers, ***cessation*** will be coded 1 for those who report having stopped smoking completely in the last year [response (d) to the measure of smoking status described above] and 0 for those who report being a current smoker [responses (a–c)].<br><br>Among past-year smokers, quit attempts will be assessed with the question: 'How many serious attempts to stop smoking have you made in the last 12 months? By serious attempt I mean you decided that you would try to make sure you never smoked again. Please include any attempt that you are currently making and please include any successful attempt made within the last year'. Those who report ***making ≥1 serious quit attempt*** in the past year will be coded 1; otherwise they will be coded 0. Among those who report making ≥1 quit attempt, ***number of past-year quit attempts*** will be analysed as a continuous variable.

Among past-year smokers who report making at least one quit attempt in the past year, use of cessation support in the most recent quit attempt will be assessed with the question: 'Which, if any, of the following did you try to help you stop smoking during the most recent serious quit attempt?'. Participants will be asked to indicate all that apply.

- ***Use of prescription medication*** will be coded 1 for those who report using varenicline, bupropion, or NRT on prescription, and 0 for those who do not report using any of these. Because varenicline was recalled in 2021 by its manufacturer, Pfizer Ltd, we will run a sensitivity analysis excluding varenicline to check that any apparent impact of the pandemic on use of prescription medication was not driven by an artefactual decline in use of varenicline due to unavailability.
- ***Use of behavioural support*** will be coded 1 for those who report attending a stop smoking group or one or more stop smoking one-to-one counselling/advice/support session(s) (either in person or remotely) or using telephone support, a website, an app, or written self-help materials, and 0 for those who do not report using any of these.
- ***Use of e-cigarettes*** will be coded 1 for those who report using e-cigarettes, and 0 for those who do not.

Uptake will be assessed by current smoking prevalence among 18-24 year olds (because any increases in this age group would largely be driven by uptake rather than relapse).

Among past-year smokers who report making at least one quit attempt in the 6-12 months before completing the survey, ***short-term relapse*** to smoking following the most recent quit attempt in this period will be coded 0 for those who do not report a subsequent quit attempt and respond that they are 'still not smoking' to the question 'How long did your most recent serious quit attempt last before you went back to smoking?'. Short-term relapse will be coded 1 for participants who report a making a subsequent quit attempt (i.e., any quit attempt in the 0-6 months prior to the survey) or report being a current smoker.

Late relapse will be assessed by current smoking prevalence among 45-65 year olds (because an increase in this age group would largely be driven by relapse rather than uptake). The upper limit for the latter group was selected to minimise any impact of increased mortality at older ages during the pandemic on smoking prevalence.

Trends, pandemic effects, and seasonality

Time will be measured in months throughout the study period (coded 1…*n*, where *n* is the total number of waves), which will allow us to account for ***underlying secular trends***. An additional variable will reflect the pre-pandemic and pandemic periods in order to identify ***step-level changes*** in the outcomes (coded 0 before the start of the pandemic [June 2017 through February 2020] and 1 after [April 2020 through August 2022]). Finally, we will include a variable to reflect the ***change in trend*** (slope) following the start of the pandemic (coded 0 before the pandemic and 1…*m* from April 2020 onwards, where *m* is the number of waves after the start of the pandemic).

To control for ***seasonality*** (month-of-year effects), the month within the year (calendar month) will be coded, from January = 1 to December = 12. This will account for any regular seasonal pattern in the outcomes of interest.

	Covariates<br><br>Analyses of smoking prevalence and quit attempts will be adjusted for age, gender, social grade and region. ***Age*** will be analysed as a continuous variable. ***Gender*** is self-reported as 'man', 'woman', or 'in another way'. Due to low numbers, those who describe their gender 'in another way' will be excluded. ***Social grade*** will be categorised as ABC1 (which includes managerial, professional and intermediate occupations) versus C2DE (which includes small employers and own-account workers, lower supervisory and technical occupations, and semi-routine and routine occupations, never workers and long-term unemployed). ***Region*** in England will be categorised as London, South, Central, and North.<br><br>Analyses of smoking cessation and use of support will be adjusted for these sociodemographic characteristics, level of dependence (because more dependent smokers tend to be less likely to quit and more likely to use support (14)), and government spending on tobacco control mass media campaigns (15–17). ***Cigarette dependence*** will be assessed with self-reported ratings of strength of urges to smoke over the past 24 hours [not at all (coded 0), slight (1), moderate (2), strong (3), very strong (4) and extremely strong (5)]. This variable will also be coded '0' for smokers who responded 'not at all' to the (separate) question: 'How much of the time have you spent with the urge to smoke?' (18). Monthly ***expenditure on tobacco control mass media campaigns*** (in £) will be obtained from the Office for Health Improvement and Disparities and adjusted for inflation. While there are other potentially relevant population-level covariates, such as affordability of e-cigarettes or prevalence of e-cigarette use, we will not be adjusting for them in this study as they could plausibly have been affected by the pandemic (19).
**Analyses**	Data will be analysed in R studio.<br><br>First, we will calculate unweighted and weighted descriptive statistics on sociodemographic and smoking characteristics. The Smoking Toolkit Study uses raking to match the sample to the population in England on the dimensions of age, social grade, region, housing tenure, ethnicity, and working status within sex (20). This profile is determined each month by combining data from the 2011 UK Census, the Office for National Statistics mid-year estimates, and the annual National Readership Survey (10). All the following analyses will be done on weighted data.<br><br>We will use segmented regression to assess the effect of the onset of the Covid-19 pandemic on each outcome. In the first instance we will use generalised additive models (GAMs). These allow the fitting of smoothing terms (e.g., cyclic cubic splines) to take seasonality into account. If there are any issues with convergence, we will use generalised linear models (GLMs). Models will be extended to generalised additive mixed models (GAMMs) if necessary to account for autocorrelation.<br><br>We will model the trend in each outcome before the pandemic (underlying secular trend), the step-level change, and change in the trend post-onset of the pandemic relative to pre-pandemic. Models will be adjusted for seasonality (modelled using a smoothing term with cyclic cubic splines specified) and covariates. A linear pre-pandemic and pandemic trend will be assumed, based on prior data (21) and the relatively short length of the time series (meaning there will be negligible differences between log-linear and linear trends). If trends appear to deviate from this assumption upon inspection of the data, we will consider using more complex trends. We will repeat these models separately by social grade (ABC1 and C2DE).

	Planned sensitivity analyses will test for pulse effects by running GAMMs with pulses lasting two and three months (coded 0 before the start of the pandemic, 1 in the two or three months after the onset of the pandemic, and 0 thereafter), assuming a constant underlying time trend. We will also rerun the model for use of prescription medication excluding varenicline, to check whether the results are affected by unavailability of this medication from mid-2021. Finally, we will rerun the models for smoking cessation and use of support excluding cigarette dependence as a covariate, because strength of urges to smoke could plausibly be affected by the Covid-19 pandemic (e.g., increased due to stress or reduced due to less exposure to others smoking) and thus adjusting for it may serve to dilute the true impact of the pandemic on these outcomes.
**Governance and ethics**	Ethical approval for the STS was granted originally by the UCL Ethics Committee (ID 0498/001). The data are not collected by UCL and are anonymised when received by UCL.
**Finance**	CRUK (PRCRPG-Nov21\100002) for STS data collection and salary for SJ.
**References**	1. Sheikh A, McMenamin J, Taylor B, Robertson C. SARS-CoV-2 Delta VOC in Scotland: demographics, risk of hospital admission, and vaccine effectiveness. The Lancet. 2021 Jun 26;397(10293):2461–2. 2. Karim SSA, Karim QA. Omicron SARS-CoV-2 variant: a new chapter in the COVID-19 pandemic. Lancet Lond Engl. 2021 Dec 11;398(10317):2126–8. 3. Jackson SE, Beard E, Angus C, Field M, Brown J. Moderators of changes in smoking, drinking and quitting behaviour associated with the first COVID-19 lockdown in England. Addiction. 2022;117(3):772–83. 4. Sarich P, Cabasag CJ, Liebermann E, Vaneckova P, Carle C, Hughes S, et al. Tobacco smoking changes during the first pre-vaccination phases of the COVID-19 pandemic: A systematic review and meta-analysis. eClinicalMedicine. 2022 May;47:101375. 5. Gravely S, Craig LV, Cummings KM, Ouimet J, Loewen R, Martin N, et al. Smokers' cognitive and behavioural reactions during the early phase of the COVID-19 pandemic: Findings from the 2020 ITC Four Country Smoking and Vaping Survey. PLOS ONE. 2021 Jun 4;16(6):e0252427. 6. Office for National Statistics. Smoking prevalence in the UK and the impact of data collection changes: 2020 [Internet]. 2021 [cited 2022 Jun 23]. Available from: https://www.ons.gov.uk/peoplepopulationandcommunity/healthandsocialcare/drugusealcoholandsmoking/bulletins/smokingprevalenceintheukandtheimpactofdatacollectionchanges/2020#smoking-prevalence-based-on-data-from-the-annual-population-survey 7. Wu P, Liu X, Fang Y, Fan B, Fuller CJ, Guan Z, et al. Alcohol Abuse/Dependence Symptoms Among Hospital Employees Exposed to a SARS Outbreak. Alcohol Alcohol. 2008 Nov 1;43(6):706–12. 8. Fidler JA, Shahab L, West O, Jarvis MJ, McEwen A, Stapleton JA, et al. 'The smoking toolkit study': a national study of smoking and smoking cessation in England. BMC Public Health. 2011 Jun 18;11:479.

9. Jackson SE, Beard E, Kujawski B, Sunyer E, Michie S, Shahab L, et al. Comparison of Trends in Self-reported Cigarette Consumption and Sales in England, 2011 to 2018. JAMA Netw Open. 2019 Aug 2;2(8):e1910161.

10. Jackson SE, Garnett C, Shahab L, Oldham M, Brown J. Association of the Covid-19 lockdown with smoking, drinking, and attempts to quit in England: an analysis of 2019-2020 data. Addiction. 2021;116(5):1233–44.

11. Heatherton TF, Kozlowski LT, Frecker RC, Rickert W, Robinson J. Measuring the Heaviness of Smoking: using self-reported time to the first cigarette of the day and number of cigarettes smoked per day. Br J Addict. 1989 Jul 1;84(7):791–800.

12. Babor TF, Higgins-Biddle JC, Saunders JB, Monteiro MG, Organization WH. AUDIT: the alcohol use disorders identification test: guidelines for use in primary health care. 2001;

13. NHS Digital. Statistics on Smoking, England 2020 [Internet]. NHS Digital. 2020 [cited 2022 Jun 23]. Available from: https://digital.nhs.uk/data-and-information/publications/statistical/statistics-on-smoking/statistics-on-smoking-england-2020

14. Beard E, West R, Michie S, Brown J. Association of prevalence of electronic cigarette use with smoking cessation and cigarette consumption in England: a time–series analysis between 2006 and 2017. Addiction. 2020;115(5):961–74.

15. Kuipers MA, Partos T, McNeill A, Beard E, Gilmore AB, West R, et al. Smokers' strategies across social grades to minimise the cost of smoking in a period with annual tax increases: evidence from a national survey in England. BMJ Open. 2019 Jun 25;9(6):e026320.

16. Chaloupka FJ, Yurekli A, Fong GT. Tobacco taxes as a tobacco control strategy. Tob Control. 2012 Mar 1;21(2):172–80.

17. Vangeli E, Stapleton J, Smit ES, Borland R, West R. Predictors of attempts to stop smoking and their success in adult general population samples: a systematic review: Predictors of quit attempts and success. Addiction. 2011 Dec;106(12):2110–21.

18. Kuipers MAG, Beard E, West R, Brown J. Associations between tobacco control mass media campaign expenditure and smoking prevalence and quitting in England: a time series analysis. Tob Control. 2018 Jul 1;27(4):455–62.

19. Beard E, Jackson SE, West R, Kuipers MAG, Brown J. Population-level predictors of changes in success rates of smoking quit attempts in England: a time series analysis. Addiction. 2020;115(2):315–25.

20. Langley T, Szatkowski L, Lewis S, McNeill A, Gilmore AB, Salway R, et al. The freeze on mass media campaigns in England: a natural experiment of the impact of tobacco control campaigns on quitting behaviour. Addict Abingdon Engl. 2014 Jun;109(6):995–1002.

21. Beard E, West R, Michie S, Brown J. Association between electronic cigarette use and changes in quit attempts, success of quit attempts, use of smoking cessation

	pharmacotherapy, and use of stop smoking services in England: time series analysis of population trends. BMJ. 2016 Sep 13;354:i4645.
