# Supplementary material for: Have there been sustained impacts of the COVID-19 pandemic on trends in smoking prevalence, uptake, quitting, use of treatment, and relapse? A monthly population study in England, 2017–2022
Source: BMC Med. 2023 Dec 14;21:474. doi: 10.1186/s12916-023-03157-2 (PMC10720231; doi:10.1186/s12916-023-03157-2)
Supplement: Supplementary file 3 — Additional file 3. Checklist. [file 12916_2023_3157_MOESM3_ESM.pdf]

Additional file 3

STROBE Statement—checklist of items that should be included in reports of observational studies

	Item No.	Recommendation	Section	Paragraph
**Title and abstract**	1	(*a*) Indicate the study's design with a commonly used term in the title or the abstract	Title	
		(*b*) Provide in the abstract an informative and balanced summary of what was done and what was found	Abstract	
**Introduction**				
Background/rationale	2	Explain the scientific background and rationale for the investigation being reported	Introduction<br>Supplementary Material	1-2
Objectives	3	State specific objectives, including any prespecified hypotheses	Introduction	3-4
**Methods**				
Study design	4	Present key elements of study design early in the paper	Introduction<br>Methods - Design	3<br>1-2
Setting	5	Describe the setting, locations, and relevant dates, including periods of recruitment, exposure, follow-up, and data collection	Methods - Design	1-3
Participants	6	(*a*) *Cohort study*—Give the eligibility criteria, and the sources and methods of selection of participants. Describe methods of follow-up<br>*Case-control study*—Give the eligibility criteria, and the sources and methods of case ascertainment and control selection. Give the rationale for the choice of cases and controls<br>*Cross-sectional study*—Give the eligibility criteria, and the sources and methods of selection of participants	Methods - Design	1-3
		(*b*) *Cohort study*—For matched studies, give matching criteria and number of exposed and unexposed<br>*Case-control study*—For matched studies, give matching criteria and the number of controls per case	N/A	
Variables	7	Clearly define all outcomes, exposures, predictors, potential confounders, and effect modifiers. Give diagnostic criteria, if applicable	Methods – Measures<br>Supplementary Material	1-3
Data sources/ measurement	8*	For each variable of interest, give sources of data and details of methods of assessment (measurement). Describe comparability of assessment methods if	Methods – Measures<br>Supplementary Material	1-3

		there is more than one group		
Bias	9	Describe any efforts to address potential sources of bias	Methods – Statistical analyses	1, 3
Study size	10	Explain how the study size was arrived at	Results	1
Quantitative variables	11	Explain how quantitative variables were handled in the analyses. If applicable, describe which groupings were chosen and why	Methods – Measures<br>Supplementary Material	1-3
Statistical methods	12	(*a*) Describe all statistical methods, including those used to control for confounding	Methods – Statistical analyses	1-4
		(*b*) Describe any methods used to examine subgroups and interactions	Methods – Statistical analyses	2
		(*c*) Explain how missing data were addressed	Methods – Statistical analyses	1
		(*d*) *Cohort study*—If applicable, explain how loss to follow-up was addressed<br>*Case-control study*—If applicable, explain how matching of cases and controls was addressed<br>*Cross-sectional study*—If applicable, describe analytical methods taking account of sampling strategy	Methods – Statistical analyses	1
		(*e*) Describe any sensitivity analyses	Methods – Statistical analyses	3-4
**Results**				
Participants	13*	(a) Report numbers of individuals at each stage of study—eg numbers potentially eligible, examined for eligibility, confirmed eligible, included in the study, completing follow-up, and analysed	Results	1
		(b) Give reasons for non-participation at each stage	Results	1
		(c) Consider use of a flow diagram	N/A	
Descriptive data	14*	(a) Give characteristics of study participants (eg demographic, clinical, social) and information on exposures and potential confounders	Table 1<br>Supplementary Table 1	
		(b) Indicate number of participants with missing data for each variable of interest	Results<br>Table 1	1<br>footnote
		(c) *Cohort study*—Summarise follow-up time (eg, average and total amount)	N/A	
Outcome data	15*	*Cohort study*—Report numbers of outcome events or summary measures over time	N/A	
		Case-control study—Report numbers in each exposure category, or summary measures of exposure	N/A	
		Cross-sectional study—Report numbers of outcome events or summary measures	Figure 1	

			Figure 2<br>Figure 3	
Main results	16	(*a*) Give unadjusted estimates and, if applicable, confounder-adjusted estimates and their precision (eg, 95% confidence interval). Make clear which confounders were adjusted for and why they were included	Table 2	
		(*b*) Report category boundaries when continuous variables were categorized	N/A	
		(*c*) If relevant, consider translating estimates of relative risk into absolute risk for a meaningful time period	N/A	
Other analyses	17	Report other analyses done—eg analyses of subgroups and interactions, and sensitivity analyses	Supplementary Material	
**Discussion**				
Key results	18	Summarise key results with reference to study objectives	Discussion	1-5
Limitations	19	Discuss limitations of the study, taking into account sources of potential bias or imprecision. Discuss both direction and magnitude of any potential bias	Discussion	6
Interpretation	20	Give a cautious overall interpretation of results considering objectives, limitations, multiplicity of analyses, results from similar studies, and other relevant evidence	Discussion	7
Generalisability	21	Discuss the generalisability (external validity) of the study results	Discussion	6

*Give information separately for cases and controls in case-control studies and, if applicable, for exposed and unexposed groups in cohort and cross-sectional studies.

Note: An Explanation and Elaboration article discusses each checklist item and gives methodological background and published examples of transparent reporting. The STROBE checklist is best used in conjunction with this article (freely available on the Web sites of PLoS Medicine at http://www.plosmedicine.org/, Annals of Internal Medicine at http://www.annals.org/, and Epidemiology at http://www.epidem.com/). Information on the STROBE Initiative is available at www.strobe-statement.org.
