# Supplementary material for: Have there been sustained impacts of the COVID-19 pandemic on trends in smoking prevalence, uptake, quitting, use of treatment, and relapse? A monthly population study in England, 2017–2022
Source: BMC Med. 2023 Dec 14;21:474. doi: 10.1186/s12916-023-03157-2 (PMC10720231; doi:10.1186/s12916-023-03157-2)
Supplement: Supplementary file 4 — Additional file 4. Measures. [file 12916_2023_3157_MOESM4_ESM.pdf]

Outcome variables

We assessed the following smoking outcomes:

- Among all adults: current smoking;
- Among 18-24 year-olds: uptake of smoking (indexed by current smoking);
- Among 45-65 year-olds: late relapse (indexed by current smoking);
- Among past-year smokers: cessation, quit attempts;
- Among past-year smokers who made ≥1 quit attempt in the past 12 months: number of quit attempts made, use of prescription medication, use of behavioural support, use of e-cigarettes;
- Among past-year smokers who made ≥1 quit attempt 6-12 months before completing the survey: medium-term abstinence.

Smoking status was assessed with the question: 'Which of the following best applies to you? Please note we are referring to cigarettes and other kinds of tobacco that you set light to and NOT electronic or 'heat-not-burn' cigarettes: (a) I smoke cigarettes (including hand-rolled) every day; (b) I smoke cigarettes (including hand-rolled), but not every day; (c) I do not smoke cigarettes at all, but I do smoke tobacco of some kind (e.g. pipe, cigar or shisha); (d) I have stopped smoking completely in the last year; (e) I stopped smoking completely more than a year ago; (f) I have never been a smoker (i.e. smoked for a year or more)'. ***Current smoking*** was coded 1 for those who reported smoking any type of tobacco [i.e. responses (a–c)] and 0 for those who reported being a former or never smoker [responses (d–f)]. Past-year smoking was coded 1 for those who reported current smoking or having stopped in the past year [responses (a–d)] and 0 for those who reported stopping more than a year ago or never smoking [responses (e–f)].

Uptake was assessed by current smoking prevalence among 18-24 year olds (because any increases in this age group would largely be driven by uptake rather than relapse).

Late relapse was assessed by current smoking prevalence among 45-65 year olds (because an increase in this age group would largely be driven by relapse rather than uptake). The upper age limit for this group was selected to minimise any impact of increased mortality at older ages during the pandemic on smoking prevalence.

Among past-year smokers, ***cessation*** was coded 1 for those who reported having stopped smoking completely in the last year [response (d) to the measure of smoking status described above] and 0 for those who reported being a current smoker [responses (a–c)].

Among past-year smokers, quit attempts were assessed with the question: 'How many serious attempts to stop smoking have you made in the last 12 months? By serious attempt I mean you decided that you would try to make sure you never smoked again. Please include any attempt that you are currently making and please include any successful attempt made within the last year'. Those who reported ***making ≥1 serious quit attempt*** in the past year were coded 1; otherwise they were coded 0.

Among those who reported making ≥1 quit attempt, ***number of past-year quit attempts*** was analysed as a continuous variable. We log-transformed the number of past-year quit attempts variable for analysis to normalise the skewed distribution and reported results as geometric means for ease of interpretation.

Among those who reported making ≥1 quit attempt, use of cessation support in the most recent quit attempt was also assessed with the question: 'Which, if any, of the following did you try to help you stop smoking during the most recent serious quit attempt?'. Participants were asked to indicate all that apply.

- ***Use of prescription medication*** was coded 1 for those who reported using varenicline, bupropion, or NRT on prescription, and 0 for those who did not report using any of these. Because varenicline was recalled in 2021 by its manufacturer, Pfizer Ltd, we ran a sensitivity analysis excluding varenicline to check that any apparent impact of the pandemic on use of prescription medication was not driven by an artefactual decline in use of varenicline due to unavailability.
- ***Use of behavioural support*** was coded 1 for those who reported attending a stop smoking group or one or more stop smoking one-to-one counselling/advice/support session(s) (either in person or remotely) or using telephone support, a website, an app, or written self-help materials, and 0 for those who did not report using any of these.
- ***Use of e-cigarettes*** was coded 1 for those who reported using e-cigarettes, and 0 for those who did not.

Among past-year smokers who reported making ≥1 quit attempt in the 6-12 months before completing the survey, ***medium-term abstinence*** following the most recent quit attempt in this period was coded 1 for those who did not report a subsequent quit attempt and responded that they were 'still not smoking' to the question 'How long did your most recent serious quit attempt last before you went back to smoking?'. Medium-term abstinence was coded 0 for participants who reported a making a subsequent quit attempt (i.e., any quit attempt in the 0-6 months prior to the survey) or reported being a current smoker.

Trends, pandemic effects, and seasonality

Time was measured in months throughout the study period (coded 1…*n*, where *n* was the total number of waves), which allowed us to account for ***underlying secular trends***. An additional variable reflected the pre-pandemic and pandemic periods in order to identify ***step-level changes*** in the outcomes (coded 0 before the start of the pandemic [June 2017 through February 2020] and 1 after [April 2020 through August 2022]). Finally, we included a variable to reflect the ***change in trend*** (slope) following the start of the pandemic (coded 0 before the pandemic and 1…*m* from April 2020 onwards, where *m* was the number of waves after the start of the pandemic). We controlled for ***seasonality*** (month-of-year effects) using cyclic cubic splines (see Statistical Analysis).

Covariates

Analyses of smoking prevalence, uptake, late relapse, and quit attempts were adjusted for age, gender, social grade and region. ***Age*** was analysed as a continuous variable. ***Gender*** was self-reported as 'man', 'woman', or 'in another way'. Due to low numbers, those who described their gender 'in another way' were excluded from analyses that adjusted for gender. ***Social grade*** was categorised as ABC1 (which includes managerial, professional and intermediate occupations) versus C2DE (which includes small employers and own-account workers, lower supervisory and technical occupations, and semi-routine and routine occupations, never workers and long-term unemployed). ***Region*** in England was categorised as London, South, Central, and North.

Analyses of smoking cessation, medium-term abstinence, and use of support were adjusted for these sociodemographic characteristics, level of dependence (because more dependent smokers tend to be less likely to quit and more likely to use support [15]), and government spending on tobacco control mass media campaigns [16-18]. ***Cigarette dependence*** was assessed with self-reported ratings of strength of urges to smoke over the past 24 hours [not at all (coded 0), slight (1), moderate (2), strong (3), very strong (4) and extremely strong (5)]. This variable was also coded '0' for smokers who responded 'not at all' to the (separate) question: 'How much of the time have you spent with the urge to smoke?' [19]. This measure has been validated and performs at least as well as the Fagerström Test of Cigarette Dependence and the Heaviness of Smoking Index in predicting the outcome of cessation while not being subject to bias due to population-level changes in cigarette consumption over the time period of the study [19]. Monthly ***expenditure on tobacco control mass media campaigns*** (in £) was obtained from the Office for Health Improvement and Disparities and adjusted for inflation. While there were other potentially relevant population-level covariates, such as affordability of e-cigarettes or prevalence of e-cigarette use, we did not adjust for them in this study as they could plausibly have been affected by the pandemic [20].
