# Supplementary material for: Have there been sustained impacts of the COVID-19 pandemic on trends in smoking prevalence, uptake, quitting, use of treatment, and relapse? A monthly population study in England, 2017–2022
Source: BMC Med. 2023 Dec 14;21:474. doi: 10.1186/s12916-023-03157-2 (PMC10720231; doi:10.1186/s12916-023-03157-2)
Supplement: Supplementary file 5 — Additional file 5: Table S1-7, Figs S1-4. Table S1 – Unweighted descriptive statistics. Table S2 – GAM results (relative risks). Table S3 – Sensitivity analysis: pulse effects. Table S4 – Sensitivity analysis: excluding dependence. Fig S1 – Quitting activity, with and without adjustment for dependence. Fig S2 – Use of support, with and without adjustment for dependence. Table S5 – Associations with cigarette dependence. Fig S3 – Cigarette dependence. Table S6 – Sensitivity analysis: cessation and quit attempts by age. Fig S4 – Cessation and quit attempts by age and social grade. Table S7 – Sensitivity analysis: excluding varenicline. [file 12916_2023_3157_MOESM5_ESM.pdf]

Table S1. Unweighted descriptive statistics

Table S2. GAM results: associations between the Covid-19 pandemic and smoking outcomes, overall and by social grade (relative risks)

<u>*Sensitivity analyses*</u>

1. Pulse effects

Table S3. Sensitivity analysis: pulse effects

2. Excluding dependence as a covariate

Table S4. Sensitivity analysis: excluding cigarette dependence as a covariate

Figure S1. Quitting activity, with and without adjustment for dependence

Figure S2. Use of support by smokers in quit attempts, with and without adjustment for dependence

3. Changes in dependence

Table S5. Associations between the Covid-19 pandemic and cigarette dependence

Figure S3. Cigarette dependence

4. By age group

Table S6. Sensitivity analysis: cessation and quit attempts by age

Figure S4. Cessation and quit attempts by age and social grade

5. Excluding varenicline from prescription medication

Table S7. Sensitivity analysis: excluding varenicline from analysis of use of prescription medication

Table S1. Unweighted descriptive statistics

	Overall	Before pandemic (June 2017-Feb 2020)	During pandemic (April 2020-Aug 2022)
N	*101 960*	*55 349*	*46 611*
Age (years)	50.2 (19.3)	49.8 (19.7)	50.7 (18.7)
18-24	12.2%	13.5%	10.6%
25-44	28.5%	28.5%	28.5%
45-65	33.7%	32.0%	35.7%
>65	25.6%	25.9%	25.2%
Gender			
Men	48.9%	49.9%	47.8%
Women	50.8%	50.0%	51.6%
In another way	0.3%	0.1%	0.6%
Social grade C2DE (less advantaged)	37.1%	39.1%	34.8%
Region in England			
London	15.8%	16.0%	15.6%
South	25.1%	23.7%	26.6%
Central	30.5%	30.9%	30.0%
North	28.6%	29.3%	27.8%
Current smoker	16.0%	16.5%	15.4%
Past-year smoker	17.6%	17.6%	17.6%
Uptake of smoking[1c]	22.0%	21.2%	23.3%
Late relapse[2c]	15.0%	16.9%	13.1%
Cigarette dependence[3a]	1.70 (1.19)	1.76 (1.14)	1.62 (1.26)
Quit in past year[3]	9.2%	6.1%	12.8%
Tried to quit in past year[3]	33.4%	30.6%	36.8%
Number of past-year quit attempts[4b]	1.43 (1.79)	1.40 (1.77)	1.45 (1.79)
Use of cessation support[4]			
Prescription medication	7.2%	7.0%	7.5%
Behavioural support	7.4%	6.4%	8.3%
E-cigarettes	30.6%	32.2%	29.1%
Monthly inflation-adjusted national tobacco control expenditure (£)	137 000 (247 000)	142 000 (237 000)	131 000 (261 000)

Data are shown as percentages or mean (SD), unless otherwise specified.

C2DE = small employers/lower supervisory/technical/semi-routine/routine/never workers/long-term unemployed.

[1] Among 18-24 year olds (*n = 12,455*). [2] Among 45-65 year olds (*n = 34,332*). [3] Among past-year smokers (*n = 17,964*). [4] Among past-year smokers who tried to quit in the past year (*n = 5,754*).

[a] Strength of urges to smoke rated on a scale from 0 (none) to 5 (extremely strong).

[b] Geometric mean.

[c] Indexed by current smoking.

There was a small amount of missing data for some variables (<0.1% gender, 2.4% cigarette dependence, 4.1% tried to quit in past year); valid percentages are shown.

Table S2. GAM results: associations between the Covid-19 pandemic and smoking outcomes, overall and by social grade (relative risks)

	Overall			Social grades ABC1			Social grades C2DE		
	**RR / *Exp. B*[*]**	**95% CI**		**RR / *Exp. B*[*]**	**95% CI**		**RR / *Exp. B*[*]**	**95% CI**	
		**Lower**	**Upper**		**Lower**	**Upper**		**Lower**	**Upper**
Current smoking[1a]									
Pre-pandemic trend (year*)	0.948	0.927	0.970	0.905	0.873	0.939	0.969	0.941	0.999
Pre vs. post step-level change	1.080	1.022	1.141	1.201	1.101	1.310	1.027	0.953	1.106
Pre vs. post Δ trend (year*)	1.052	1.014	1.090	1.145	1.083	1.211	1.003	0.955	1.054
Current smoking among young adults[2a]									
Pre-pandemic trend (year*)	0.895	0.846	0.946	0.876	0.809	0.948	0.909	0.838	0.986
Pre vs. post step-level change	1.349	1.177	1.547	1.369	1.127	1.663	1.342	1.101	1.636
Pre vs. post Δ trend (year*)	1.072	0.982	1.171	1.138	1.004	1.290	1.018	0.896	1.158
Current smoking among middle-aged adults[3a]									
Pre-pandemic trend (year*)	0.943	0.907	0.981	0.883	0.828	0.942	0.974	0.924	1.026
Pre vs. post step-level change	0.864	0.781	0.956	1.069	0.909	1.257	0.776	0.674	0.893
Pre vs. post Δ trend (year*)	1.096	1.025	1.172	1.171	1.055	1.300	1.058	0.964	1.162
Cessation[4b]									
Pre-pandemic trend (year*)	0.839	0.761	0.926	0.926	0.820	1.047	0.755	0.645	0.884
Pre vs. post step-level change	2.204	1.794	2.709	1.770	1.371	2.286	2.742	1.969	3.818
Pre vs. post Δ trend (year*)	1.219	1.079	1.379	1.032	0.882	1.207	1.454	1.200	1.762
Past-year quit attempt[4a]									
Pre-pandemic trend (year*)	0.918	0.883	0.955	0.933	0.883	0.985	0.910	0.862	0.962
Pre vs. post step-level change	1.417	1.297	1.547	1.349	1.193	1.527	1.460	1.290	1.654
Pre vs. post Δ trend (year*)	1.074	1.016	1.136	1.018	0.940	1.102	1.115	1.031	1.206
Number of past-year quit attempts[5a**]									
Pre-pandemic trend (year*)	1.030	1.003	1.058	1.043	1.006	1.081	1.020	0.981	1.061
Pre vs. post step-level change	0.970	0.911	1.032	0.893	0.821	0.971	1.034	0.944	1.133
Pre vs. post Δ trend (year*)	0.997	0.958	1.038	1.023	0.969	1.081	0.981	0.926	1.040

Table continued on next page.

Table S2. *(continued)*

	Overall			Social grades ABC1			Social grades C2DE		
	RR /	95% CI		RR /	95% CI		RR /	95% CI	
	Exp. B*	Lower	Upper	*Exp. B**	Lower	Upper	*Exp. B**	Lower	Upper
Use of prescription medication[5b]									
Pre-pandemic trend (year*)	1.052	0.890	1.244	1.216	0.933	1.584	1.013	0.811	1.266
Pre vs. post step-level change	1.286	0.887	1.865	0.769	0.415	1.403	1.499	0.925	2.428
Pre vs. post Δ trend (year*)	0.845	0.662	1.080	0.860	0.580	1.275	0.826	0.601	1.135
Use of behavioural support[5b]									
Pre-pandemic trend (year*)	0.838	0.696	1.009	0.790	0.608	1.026	0.876	0.670	1.143
Pre vs. post step-level change	2.330	1.553	3.496	2.204	1.212	4.008	2.363	1.342	4.161
Pre vs. post Δ trend (year*)	1.032	0.804	1.326	1.118	0.777	1.607	0.980	0.690	1.391
Use of e-cigarettes[5b]									
Pre-pandemic trend (year*)	0.959	0.898	1.025	0.991	0.898	1.093	0.941	0.859	1.030
Pre vs. post step-level change	0.788	0.666	0.932	0.711	0.552	0.915	0.841	0.670	1.056
Pre vs. post Δ trend (year*)	1.232	1.111	1.365	1.241	1.061	1.452	1.225	1.067	1.408

ABC1 = managerial/professional/intermediate; C2DE = small employers/lower supervisory/technical/semi-routine/routine/never workers/long-term unemployed; CI = confidence interval; RR = relative risk (categorical outcomes); Exp. B = exponential beta (continuous outcomes).

[1] Among all adults. [2] Among 18-24 year-olds. [3] Among 45-65 year-olds. [4] Among past-year smokers. [5] Among past-year smokers who made ≥1 quit attempt in the past 12 months.

[a] Adjusted for seasonality, age, gender, social grade, and region. [b] Adjusted for seasonality, age, gender, social grade, region, cigarette dependence, and national expenditure on tobacco control mass media campaigns.

* Monthly trends were analysed. We multiplied the coefficients by 12 to derive annual trends.

** Number of quit attempts was analysed as a continuous variable and was log-transformed for analysis. Results are reported as exponentiated coefficients.

Pre vs. post step-level change is the step-level change associated with the start of the pandemic. Pre vs. post Δ trend is the change in trend (slope) following the start of the pandemic.

Table S3. Sensitivity analysis: pulse effects

		95% CI	
	% change	Lower	Upper
Current smoking[1a]			
2-month pulse	2.9	-4.4	10.8
3-month pulse	-0.2	-6.4	6.3
Current smoking among young adults[2a]			
2-month pulse	15.2	-3.1	37.0
3-month pulse	5.9	-9.3	23.6
Current smoking among middle-aged adults[3a]			
2-month pulse	-6.8	-19.3	7.6
3-month pulse	-10.6	-20.8	0.9
Cessation[4b]			
2-month pulse	39.2	12.9	71.7
3-month pulse	38.2	16.2	64.4
Past-year quit attempt[4a]			
2-month pulse	11.3	0.1	23.8
3-month pulse	13.0	3.4	23.5
Number of past-year quit attempts[5a*]			
2-month pulse	-0.3	-7.8	7.8
3-month pulse	0.0	-6.3	6.7
Use of prescription medication[5b]			
2-month pulse	1.9	-38.6	69.3
3-month pulse	34.9	-5.7	93.0
Use of behavioural support[5b]			
2-month pulse	108.1	31.7	228.8
3-month pulse	86.6	29.6	168.5
Use of e-cigarettes[5b]			
2-month pulse	-25.7	-41.6	-5.6
3-month pulse	-29.4	-42.5	-13.3

[1] Among all adults. [2] Among 18-24 year-olds. [3] Among 45-65 year-olds. [4] Among past-year smokers. [5] Among past-year smokers who made ≥1 quit attempt in the past 12 months.
[a] Adjusted for underlying trend, seasonality, age, gender, social grade, and region. [b] Adjusted for underlying trend, seasonality, age, gender, social grade, region, cigarette dependence, and national expenditure on tobacco control mass media campaigns.
* Number of quit attempts was analysed as a continuous variable and was log-transformed for analysis. Results are reported as percentage changes ((relative risk – 1)*100).

Table S4. Sensitivity analysis: excluding cigarette dependence as a covariate

	% change	95% CI Lower	95% CI Upper
Cessation[1a]			
Pre-pandemic trend (year*)	-17.2	-25.2	-8.2
Pre vs. post step-level change	154.4	104.8	216.1
Pre vs. post Δ trend (year*)	24.6	9.3	42.1
Use of prescription medication[2b]			
Pre-pandemic trend (year*)	5.8	-10.8	25.3
Pre vs. post step-level change	18.9	-18.4	73.5
Pre vs. post Δ trend (year*)	-15.0	-33.4	8.6
Use of behavioural support[2b]			
Pre-pandemic trend (year*)	-16.4	-30.6	0.5
Pre vs. post step-level change	127.0	51.5	240.1
Pre vs. post Δ trend (year*)	3.6	-19.2	33.0
Use of e-cigarettes[2b]			
Pre-pandemic trend (year*)	-4.0	-10.2	2.6
Pre vs. post step-level change	-22.4	-34.3	-8.3
Pre vs. post Δ trend (year*)	23.2	11.2	36.5

CI = confidence interval.

[1] Among past-year smokers. [2] Among past-year smokers who made ≥1 quit attempt in the past 12 months.

[a] Adjusted for seasonality, age, gender, social grade, region, and national expenditure on tobacco control mass media campaigns.

* Monthly trends were analysed. We multiplied the coefficients by 12 to derive annual trends.

Results are reported as percentage changes ((relative risk – 1)*100).

Pre vs post step-level change is the step-level change associated with the start of the pandemic. Pre vs. post Δ trend is the change in trend (slope) following the start of the pandemic (where the 95% CI does not overlap zero, this indicates trends differed significantly between the two time periods).

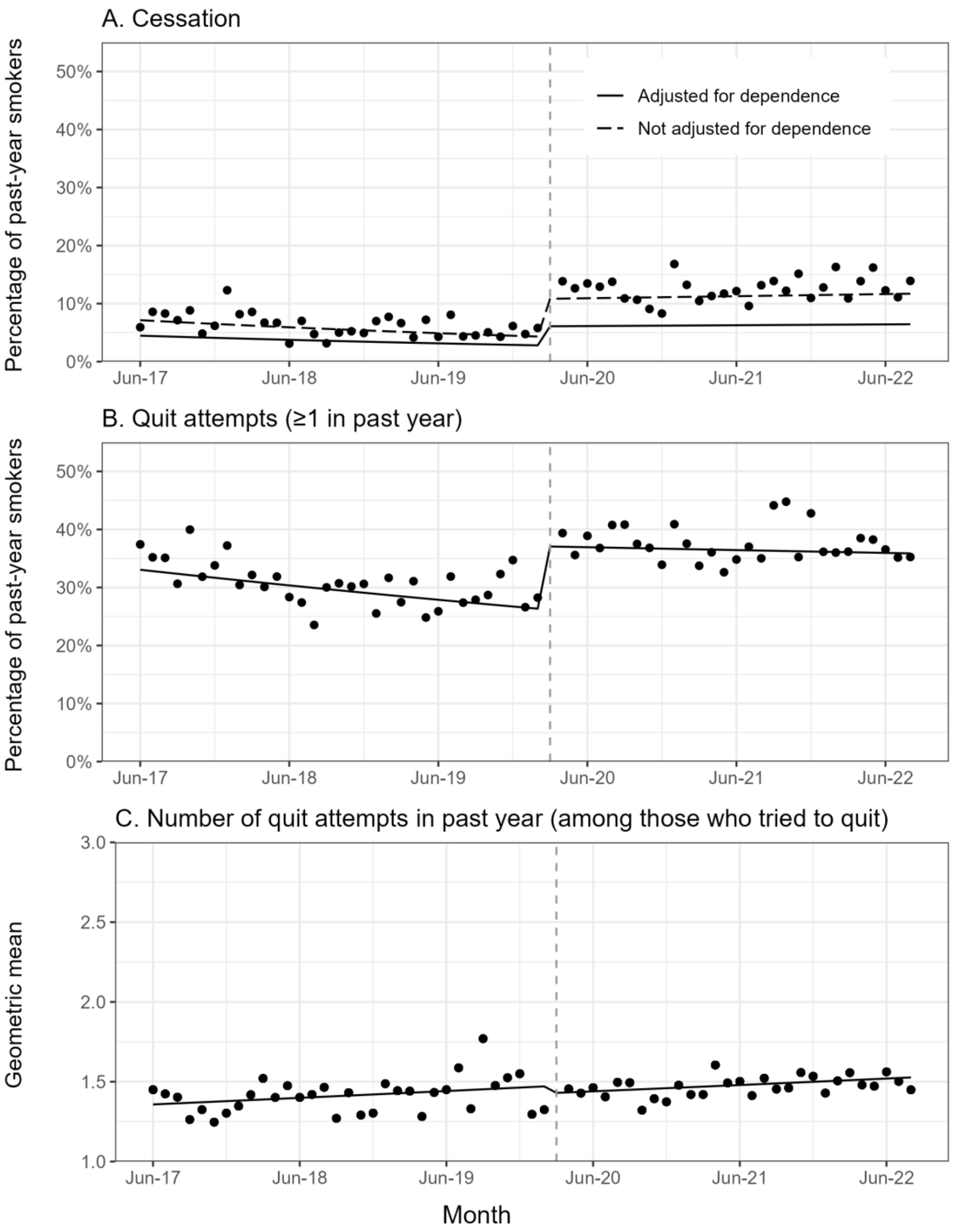

Figure S1. Quitting activity, with and without adjustment for dependence. Panels show trends in the prevalence of (A) cessation and (B) making at least one quit attempt in the past year among past-year smokers (*unweighted n = 17,964*), and (C) the weighted geometric mean number of past-year quit attempts among past-year smokers who made at least one quit attempt (*unweighted n = 5,754*), June 2017 to August 2022. Lines represent modelled weighted prevalence (or means) over the study period, adjusted for covariates. Points represent raw weighted prevalence (or means) by month. The vertical dashed line indicates the timing of the start of the Covid-19 pandemic in England (March 2020).

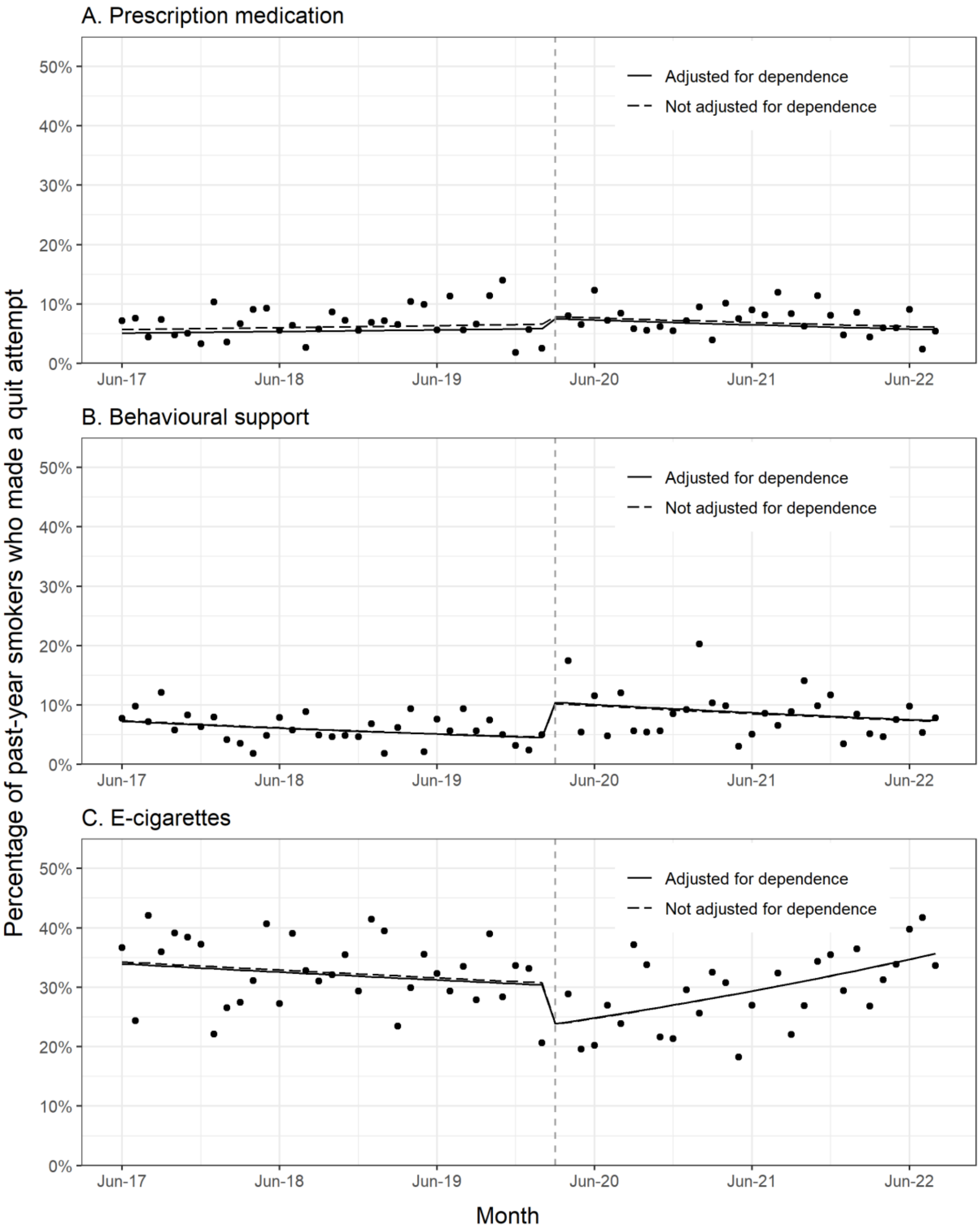

Figure S2. Use of support by smokers in quit attempts, with and without adjustment for dependence. Panels show trends in the prevalence of use of (A) prescription medication, (B) behavioural support, and (C) e-cigarettes in the most recent quit attempt among past-year smokers who made a least one quit attempt (*unweighted n = 5,754*), June 2017 to August 2022. Lines represent modelled weighted prevalence over the study period, adjusted for covariates. Points represent raw weighted prevalence by month. The vertical dashed line indicates the timing of the start of the Covid-19 pandemic in England (March 2020).

Table S5. Associations between the Covid-19 pandemic and cigarette dependence

		95% CI	
	B	**Lower**	**Upper**
Cigarette dependence[1a]			
Pre-pandemic trend (year*)	-0.003	-0.033	0.027
Pre vs. post step-level change	-0.107	-0.179	-0.035
Pre vs. post Δ trend (year*)	0.002	-0.046	0.049

CI = confidence interval.
[1] Among past-year smokers.
[a] Adjusted for seasonality, age, gender, social grade, and region.
* Monthly trends were analysed. We multiplied the coefficients by 12 to derive annual trends.
Pre vs. post step-level change is the step-level change associated with the start of the pandemic. Pre vs. post Δ trend is the change in trend (slope) following the start of the pandemic (where the 95% CI does not overlap zero, this indicates trends differed significantly between the two time periods).

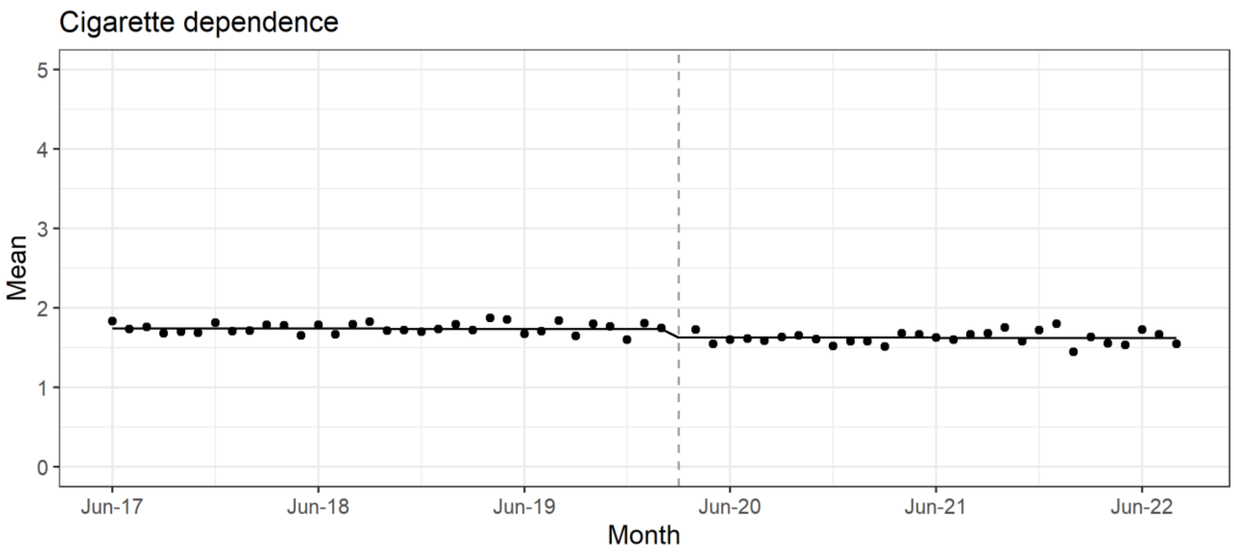

Figure S3. Cigarette dependence. Panels show trends in mean cigarette dependence among past-year smokers (*unweighted n = 17,964*), June 2017 to August 2022. Lines represent the modelled weighted mean over the study period, adjusted for covariates. Points represent raw weighted means by month. The vertical dashed line indicates the timing of the start of the Covid-19 pandemic in England (March 2020).

Table S6. Sensitivity analysis: cessation and quit attempts by age

	Age 18-24y								
	Overall			Social grades ABC1 (more advantaged)			Social grades C2DE (less advantaged)		
	% change	95% CI Lower	95% CI Upper	% change	95% CI Lower	95% CI Upper	% change	95% CI Lower	95% CI Upper
Cessation[1b]									
Pre-pandemic trend (year*)	3.9	-17.9	31.4	-4.6	-28.3	27.0	8.1	-27.0	59.9
Pre vs. post step-level change	110.7	32.8	234.4	134.8	30.5	322.4	126.4	7.3	377.4
Pre vs. post Δ trend (year*)	2.9	-22.3	36.4	2.3	-28.4	46.2	3.1	-34.4	62.1
Past-year quit attempt[1a]									
Pre-pandemic trend (year*)	-6.0	-14.4	3.2	-8.4	-19.2	3.8	-3.8	-16.4	10.5
Pre vs. post step-level change	90.8	57.0	131.9	84.5	41.2	141.1	94.5	46.7	157.9
Pre vs. post Δ trend (year*)	1.1	-10.4	14.1	1.8	-13.9	20.3	0.0	-16.0	19.0

	Age ≥25y								
	Overall			Social grades ABC1 (more advantaged)			Social grades C2DE (less advantaged)		
	% change	95% CI Lower	95% CI Upper	% change	95% CI Lower	95% CI Upper	% change	95% CI Lower	95% CI Upper
Cessation[1b]									
Pre-pandemic trend (year*)	-19.7	-27.9	-10.6	-9.3	-20.6	3.5	-30.1	-41.2	-17.0
Pre vs. post step-level change	116.7	72.4	172.2	67.8	26.7	122.2	177.7	92.1	301.3
Pre vs. post Δ trend (year*)	26.1	10.1	44.4	3.8	-12.6	23.3	59.7	29.2	97.3
Past-year quit attempt[1a]									
Pre-pandemic trend (year*)	-9.0	-12.8	-5.0	-6.3	-11.8	-0.5	-10.7	-15.9	-5.2
Pre vs. post step-level change	31.5	19.1	45.2	23.1	7.0	41.5	36.6	18.9	57.0
Pre vs. post Δ trend (year*)	9.6	2.9	16.8	2.1	-6.7	11.7	15.2	5.5	25.9

[1] Among past-year smokers.
[a] Adjusted for seasonality, age, gender, social grade, region, cigarette dependence, and national expenditure on tobacco control mass media campaigns. [b] Adjusted for seasonality, age, gender, social grade, and region.
* Monthly trends were analysed. We multiplied the coefficients by 12 to derive annual trends.
Results are reported as percentage changes ((relative risk – 1)*100).
Pre vs. post step-level change is the step-level change associated with the start of the pandemic. Pre vs. post Δ trend is the change in trend (slope) following the start of the pandemic (where the 95% CI does not overlap zero, this indicates trends differed significantly between the two time periods).

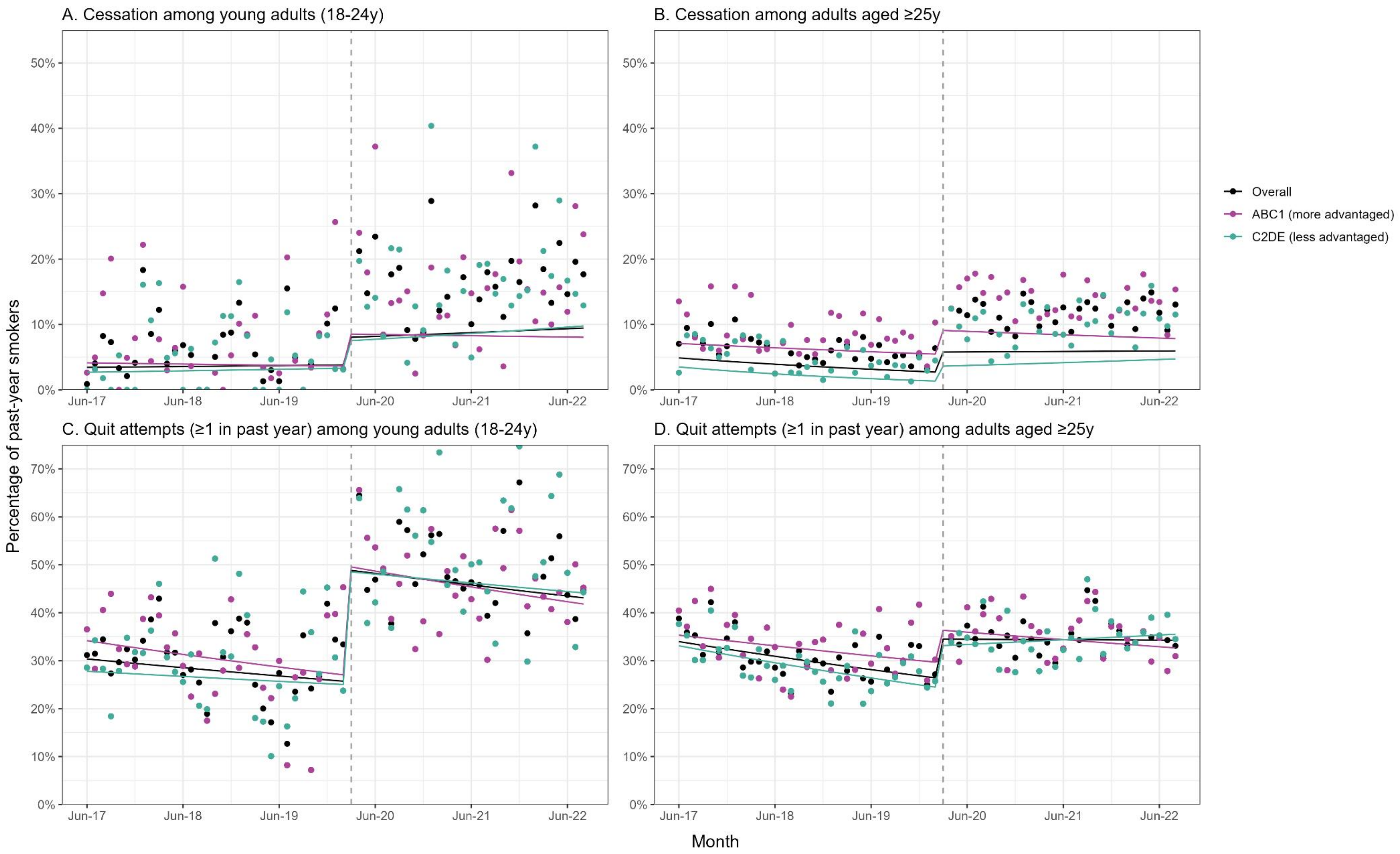

Figure S4. Cessation and quit attempts by age and social grade. Panels show trends in the prevalence of cessation and making at least one quit attempt in the past year among past-year smokers aged 18-24 years (*unweighted n: overall = 3,069, ABC1 = 1,664, C2DE = 1,405*) and those aged ≥25 years (*unweighted n: overall = 14,895, ABC1 = 7,138, C2DE = 7,757*), June 2017 to August 2022. Lines represent modelled weighted prevalence (or means) over the study period, adjusted for covariates. Points represent unadjusted weighted prevalence (or means) by month. The vertical dashed line indicates the timing of the start of the Covid-19 pandemic in England (March 2020). ABC1 = managerial/professional/intermediate; C2DE = small employers/lower supervisory/technical/semi-routine/routine/never workers/long-term unemployed.

Table S7. Sensitivity analysis: excluding varenicline from analysis of use of prescription medication

	% change	95% CI Lower	95% CI Upper
Use of prescription medication[1a]			
Pre-pandemic trend (year*)	8.6	-14.4	37.9
Pre vs. post step-level change	-3.8	-43.5	63.7
Pre vs. post Δ trend (year*)	13.6	-18.9	59.0

CI = confidence interval.

[1] Among past-year smokers who made ≥1 quit attempt in the past 12 months.

[a] Adjusted for seasonality, age, gender, social grade, region, cigarette dependence, and national expenditure on tobacco control mass media campaigns.

* Monthly trends were analysed. We multiplied the coefficients by 12 to derive annual trends. Results are reported as percentage changes ((relative risk – 1)*100).

Pre vs. post step-level change is the step-level change associated with the start of the pandemic. Pre vs. post Δ trend is the change in trend (slope) following the start of the pandemic (where the 95% CI does not overlap zero, this indicates trends differed significantly between the two time periods).
